# Mathematical modeling of *JAK2V617F* clonal expansion in a general population cohort

**DOI:** 10.1073/pnas.2507773123

**Published:** 2026-06-17

**Authors:** Jordan Snyder, Morten Andersen, Johanne Gudmand-Høyer, Morten Kranker Larsen, Vibe Skov, Lasse Kjær, Christina Schjellerup Eickhardt-Dalbøge, Trine A. Knudsen, Christina Ellervik, Hans C. Hasselbalch, Johnny T. Ottesen, Thomas Stiehl

**Affiliations:** ^a^ Department of Science and Environment, Centre for Mathematical Modeling - Human Health and Disease, IMFUFA, Roskilde University, Roskilde 4000, Denmark; ^b^ Department of Mathematics, North Carolina State University, Raleigh, NC 27607; ^c^ Department of Hematology, Zealand University Hospital, Roskilde 4000, Denmark; ^d^ Department of Clinical Medicine, Faculty of Health and Medical Sciences, University of Copenhagen, Copenhagen 2200, Denmark; ^e^ Department of Laboratory Medicine, Boston Children’s Hospital, Boston, MA 02115; ^f^Department of Pathology, Harvard Medical School, Boston, MA 02115; ^g^Department of Clinical Biochemistry, Zealand University Hospital, Køge 4600, Denmark; ^h^ Institue for Computational Biomedicine and Disease Modelling with focus on Phase Transitions between Phenotypes, Rheinisch-Westfälische Technische Hochschule Aachen University, Aachen 52074, Germany; ^i^ Center for Computational Life Sciences, Rheinisch-Westfälische Technische Hochschule Aachen University, Aachen 52074, Germany

**Keywords:** myeloproliferative neoplasms, mathematical modeling, early detection

## Abstract

The Philadelphia chromosome-negative myeloproliferative neoplasms (MPNs) are a group of blood cancers characterized by overproduction of one or more types of blood cells, which can lead to thrombosis and other complications. MPNs develop slowly and are driven by a relatively small set of mutations in the hematopoietic stem cells (HSCs). Their slow development (over the course of decades) affords a unique opportunity to study their onset, but until recently few data have been available from individuals not yet showing overt disease. Thanks to the ambitious Danish General Suburban Population Study conducted in suburban Zealand, Denmark, we have identified a (n=67) cohort of individuals harboring the most common driver mutation in MPN (namely *JAK2V617F*) and have obtained follow-up measurements of their variant allele fraction (VAF) spanning over 10 y. We show that these data are consistent with a Moran model governing the competition between healthy and mutated HSCs, and estimate the selective advantage of the mutant clone for each individual. Notably, we find that for many individuals, the change in VAF over many years is statistically consistent with zero, or even negative, selective advantage. This is in contrast to prior studies that have focused on patients diagnosed with overt MPN disease, in whom the mutant cells are almost always found to outcompete the healthy cells. Our results have implications for our understanding of the very early phases of MPN disease, and may contribute to early detection and personalized prediction of disease progression.

## Significance

This study leverages the Danish General Suburban Population Study to track the progression of the cancer-associated mutation *JAK2V617F* in individuals from the general population before the onset of disease. Unlike previous research on diagnosed patients, this work reveals that many individuals with the mutation show no clonal expansion, or even show clonal contraction, challenging existing assumptions about early myeloproliferative neoplasms development. These findings offer insights into the initial phases of the disease and may help improve patient outcomes through tailored monitoring and intervention strategies.

## Introduction

### MPN Background.

The Philadelphia Chromosome-negative myeloproliferative neoplasms (MPNs) are a group of blood cancers marked by overproduction of myeloid blood cells, which include erythrocytes (red blood cells), certain subtypes of leukocytes (white blood cells), and platelets. MPNs are commonly divided into specific subtypes of essential thrombocythemia (ET), polycythemia vera (PV), and primary myelofibrosis (PMF) (1). Most cases of MPN are driven by the *JAK2V617F* mutation in hematopoietic stem cells (HSCs) (2, 3), which is found in approximately 92% of PV patients, 55% of ET patients, and 50% of PMF patients (1). The *JAK2V617F* mutation has been estimated to be acquired early in life, possibly even in utero (4). One of the most important outstanding questions regarding MPN is how a single driver mutation, *JAK2V617F*, can give rise to multiple distinct disease phenotypes and profiles of disease progression (5–8).

Among MPN patients with an identified driver mutation (such as *JAK2V617F*, but also mutations in *CALR* or *MPL*), one can consider the variant allele fraction (VAF) as a marker of disease burden and a key way to quantify progression and response to treatment (9–13). Observation of driver mutations in MPNs naturally raises the question of when those mutations first occurred, and how they expand on the way to disease onset. This question is far from answered but has recently been addressed by a case study (14) as well as by mathematical modeling (15).

### Problem Statement.

In this study we aim to quantify the self-renewal advantage of the *JAK2V617F* mutant stem cells over wild type, toward a better understanding of the factors that contribute to MPN disease progression. We hypothesize that individuals who show a rapid increase in *JAK2V617F* VAF are more likely to transition to MPN disease. By quantifying the rate of change of VAF and relating it to MPN disease progression, we hope to deepen our understanding of the dynamics of the MPN driver clones in early predisease stages and thereby pave the way for early screening and disease prevention.

### Prior Work.

It has been known at least since 2005 that *JAK2V617F* is a driver mutation for MPN (2, 3), and since then much work has been done to understand the precise mechanism by which it leads to disease. It has been identified in a large majority of MPN patients, especially those with PV (1). It is important to emphasize that the link between *JAK2V617F* and MPN is not mere association, but is at least partially understood mechanistically (2) and has been demonstrated experimentally in a mouse model (16).

In contrast, it has been observed that MPN-associated mutations, including driver mutations, can occur in the blood without hematological malignancy, a phenomenon termed Clonal Hematopoiesis of Indeterminate Potential (CHIP) by Steensma et al. (17). Steensma et al. discuss CHIP as a clinical category and its relationship to diagnosis of various hematological diseases, as well as overall health outcomes.

The clinical relevance of *JAK2V617F* VAF has been the subject of several studies. Perricone et al. studied a cohort that was screened for *JAK2V617F* on suspicion of hematological malignancy, and found that even very low VAFs were associated with eventual MPN diagnosis (18). Notably, that study also observed spontaneous decline of *JAK2V617F* VAF in four individuals. In another study, Limvorapitak et al. did a retrospective analysis of all 5,079 patients who had been screened for *JAK2V617F* from 2010 to 2015 in British Columbia, Canada (19). Of these, 189 *JAK2V617F*-positive individuals were investigated, of whom 100 had VAF between 2-10% and 89 had VAF <2%. The key finding of that study was an overall similar prognosis in terms of survival and development of complications for the VAF <2% cohort and the VAF 2-10% cohort.

Information on *JAK2V617F* in the general population, as opposed to cohorts screened for mutation on suspicion of hematological malignancy, are relatively rare. One major effort in this direction is the Copenhagen General Population Study, which screened blood samples from 49,488 Danish citizens in the years 2003–2008 and found 0.1% (n=68 individuals) to harbor *JAK2V617F* mutation using an assay with a lower limit of detection of 0.8% variant allele fraction (20). Further, they found that *JAK2V617F* mutation was significantly associated with hematological malignancy, specifically hematological cancer, as well as ischemic heart disease and venous thromboembolism. The same individuals found to be mutation-positive were invited for follow-up screening in 2012, of whom 26 could be reexamined. Of these, 18 were found to have MPN disease. Among those with MPN disease diagnosed at follow-up, there was found to be a significant increase of VAF with time, while no significant increase was found among those without MPN disease at follow-up (21). The nonincrease of VAF among a significant subset of mutation-harboring individuals suggests that *JAK2V617F* mutation may remain at subclinical levels in many individuals for a long time, calling for further research to understand how and why this is the case.

More recently, the Danish General Suburban Population Study (GESUS), which enrolled 19,958 individuals in the years 2010–2013, analyzed blood samples for *JAK2V617F* with a much more sensitive assay (lower limit of detection 0.009%) and found a much higher prevalence at 3.1% (n=613), with the large majority having VAF less than 1% (n=507) (22). The stark contrast between the prevalence of *JAK2V617F* mutation and MPN disease [annual incidence on the order of 1 per 100,000 (23)], combined with the preponderance of low VAFs, imply that expansion of the *JAK2V617F* clone is far from guaranteed. In contrast, there must be many cases of *JAK2V617F* mutation that exhibit low or no clonal expansion, or even spontaneous reduction. It remains to quantify this phenomenon precisely.

Recently, Hermange et al. have applied a branching process model together with sophisticated Bayesian inference techniques to gain information on the timing of first mutation and expansion rate in MPNs (15). Their model includes (stochastic) exponential growth in the number of mutant stem cells while the number of wild type stem cells stays fixed, and makes use of clonal fraction (CF) measurements taken among progenitor cells. The key parameters they estimate are the rate of exponential growth in the mutant clone and the time of first mutation. They conclude that there is a possibility that *JAK2V617F* mutation was acquired in fetal life, and that the expansion rate of *CALR* mutated stem cells tends to be higher than that for *JAK2V617F* mutated stem cells. The dataset they use consists of 26 MPN patients, with a single CF measurement per patient. The nature of the data and the model used imply an assumption that the dynamics of the mutant clone are the same since first mutation, and moreover are the same for all subjects (with differences accounted for by random variation). This is in contrast with the data of the present study, which includes several time points per individual, allowing for inference on a per-individual basis.

### Our Approach.

The Danish General Suburban Population Study (GESUS) (24) was able to identify many citizens with the *JAK2V617F* mutation but no disease as defined by the 2016 WHO classification of myeloid neoplasms and acute leukemia (25) [i.e. CHIP (17)], and acquire long-term follow-up data (26). We specifically focus on a group of 67 individuals that we term the “>1% *JAK2V617F* GESUS cohort” with *JAK2V617F* VAF greater than 1% at baseline and for whom at least one follow-up measurement is available (see *Materials and Methods* for a detailed description of subject selection and data collection). These data give us the unique opportunity to study *JAK2V617F* clonal expansion starting from extremely low VAFs, and in individuals not necessarily showing any hematological malignancy. This gives us a clearer picture than ever before of the dynamics of the *JAK2V617F* clone. We view these data through the lens of a mechanistic mathematical model at the stem cell level that incorporates a proliferative advantage on behalf of mutant stem cells, as well as a finite carrying capacity of stem cells in the body. Having long-term longitudinal data allows us to quantify disease dynamics on a per-individual basis, and, in particular, allows us to observe and quantify spontaneous decline of VAF, in contrast with prior studies undertaken with only one measurement per subject (15).

## Results

To understand the expansion of the *JAK2V617F* mutation within the stem cell niche, we use a mathematical model that incorporates two key factors: that mutation can affect a cell’s propensity to replicate and/or die and that there is a fixed capacity for stem cells in the human body. The existence of a finite carrying capacity for stem cells is an important feature in our model, since it leads to saturation instead of unlimited exponential growth, as would be predicted by some simpler models. The actual size of this carrying capacity, N, i.e. the total number of stem cells in the human body, is a topic of active research; recent estimates place it on the order of $1\times 10^{4}-2\times 10^{5}$ (27–32). We assume a value of $10^{5}$, but have also carried out the same analysis using values over the entire range $1\times 10^{4}-2\times 10^{5}$. For a detailed discussion of the robustness of our results to the choice of N in our model, see *SI Appendix*, section 2.2.

**Fig. 1.**
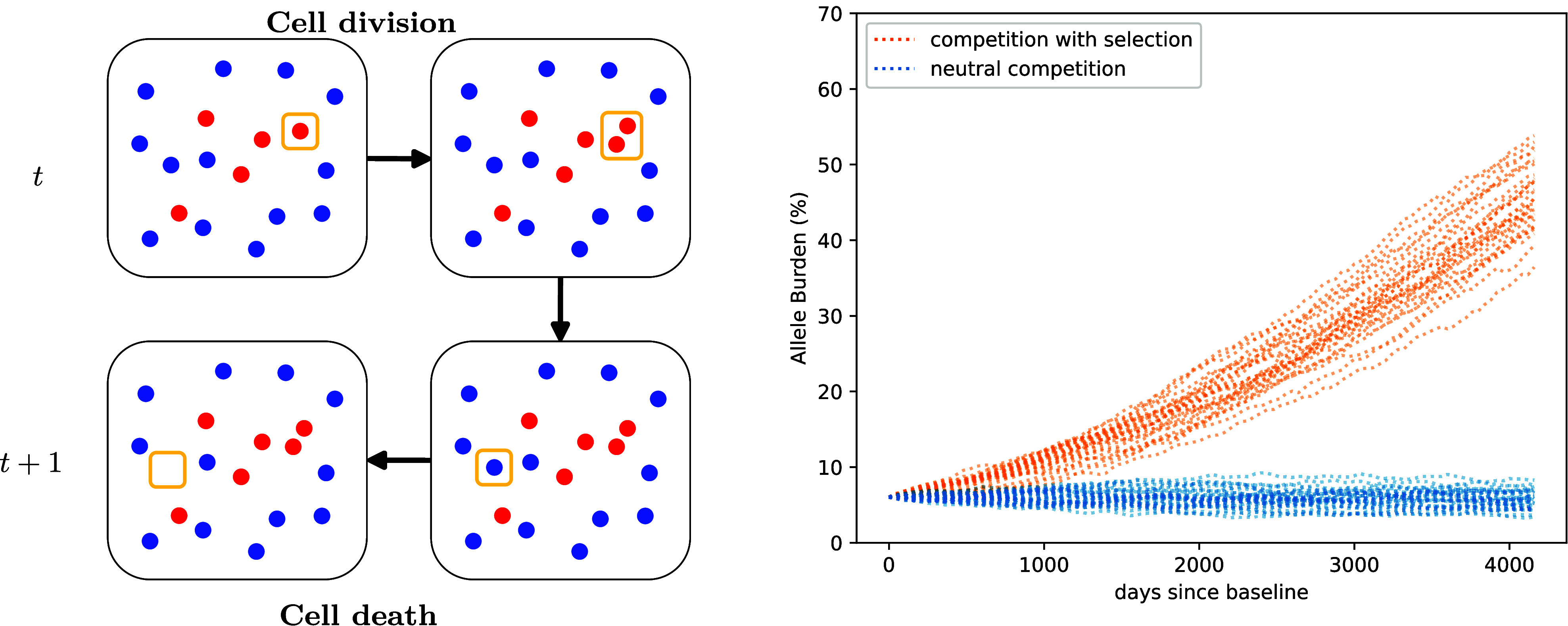
(*Left*) Diagram illustrating one time step in the Moran process. Blue dots represent normal stem cells, and red dots represent mutated stem cell. Each step of the process consists of one cell being selected at random to divide, replicating itself, and one cell being selected to die. The total number of cells remains constant, but the number of mutated cells can change. (*Right*) Two ensembles of trajectories illustrating the random development of the fraction of mutated cells over time. In blue are trajectories with s=0, i.e. no selective advantage, and in orange are trajectories with s>0.

To account for random variability in the number of mutated stem cells over time, we use a stochastic process model known as a Moran process (33). In the Moran process, we imagine a population of N cells, of which some are mutant (*JAK2V617F* in our case) and some are wild type. At each step of the process, one cell is selected to replicate, and another is selected to die (Fig. 1). In this way, the number of mutant cells can change while the total number of cells remains fixed.

To model the fact that mutant cells may tend to divide at a different rate than normal cells, we introduce a free parameter s that quantifies the self-renewal advantage of *JAK2V617F* mutant cells as compared to wild type. The value of s can be directly interpreted as the relative advantage of mutant cells over normal cells in terms of self-replication rate, for example a value of s=0.2 would mean that *JAK2V617F* mutant stem cells, on average, self-replicate 20% faster than wild type stem cells. Equivalently, the maximum rate of increase of VAF is given by $s/T_{g}$, where $T_{g}$ is the typical timescale of HSC division, which current estimates place between 23 wk (161 d) to 67 wk (469 d) (34, 35). Throughout, we assume $T_{g}=200$ d, but we obtain comparable results with $T_{g}=100$ d (*SI Appendix*, section 2.2). Our focus is to infer the self-renewal advantage (i.e. the value of s) for each subject in the >1% *JAK2V617F* GESUS cohort.

In the case s=0, mutant cells divide at the same rate as wild type cells, and we refer to this case as neutral drift. Under neutral drift, the expected number of mutant stem cells is constant over time, while the actual number of cells can increase or decrease at random. The *Right* panel of Fig. 1 depicts the effect of changing s on the process, showing that increasing s above 0 tends to make the VAF increase.

Here we present results from fitting the Moran model to data from GESUS using Approximate Bayesian Computation - Sequential Monte Carlo (ABC-SMC), a simulation-based inference method (36). Note, importantly, that the model operates in terms of the number of mutated stem cells, while measurements are of the number of variant alleles in peripheral blood. To account for the cascade of differentiation stages between stem cells and mature cells, we follow a similar approach to refs. 15 and 37 and suppose that when a *JAK2V617F* stem cell divides, it has a probability $p_{0}$ of differentiating into two progenitor cells, a probability $p_{1}$ of undergoing asymmetric division (i.e. producing one stem cell and one progenitor cell) and a probability $p_{2}$ of symmetric self-renewal, i.e. producing two stem cells. The quantity $1-\Delta =2p_{0}+p_{1}$ then gives the rate of production of *JAK2V617F* progenitor cells from stem cells, and prior work estimates this quantity at 1−0.017=0.983 (15). Next, we suppose that the *JAK2V617F* mutation confers a propensity to overproduce mature cells from progenitor cells by a factor $k_{m}=2$ (38). If we suppose that a fraction z∈[0,1] of the *JAK2V617F* mutant stem cells are heterozygous, this leads to a predicted VAF of

[1] $$\begin{array}{c} \text{VAF}\left(t\right)=\frac{\left(z+2\left(1-z\right)\right)\left(1-\Delta \right)k_{m}X\left(t\right)}{2\left(\left(N-X\left(t\right)\right)+\left(1-\Delta \right)k_{m}X\left(t\right)\right)}, \end{array}$$

where X(t) denotes the number of *JAK2V617F* mutant stem cells at time t. Here the numerator represents the number of *JAK2V617F* mutant alleles in the peripheral blood (twice as many per homozygous stem cell as per heterozygous stem cell) and the denominator represents the total number of alleles (note that N−X(t) is the number of WT stem cells at time t). Note that if z=0 (i.e. all mutant cells are homozygous) and $\left(1-\Delta \right)k_{m}=1$ then VAF(t)=X(t)/N, i.e. the VAF is equal to the clonal fraction among stem cells. We take the values $k_{m}=2$, Δ=0.017 (15) and z=0.5. For a discussion of the sensitivity of our results to these parameters, see *SI Appendix*, section 2.3.

**Fig. 2.**
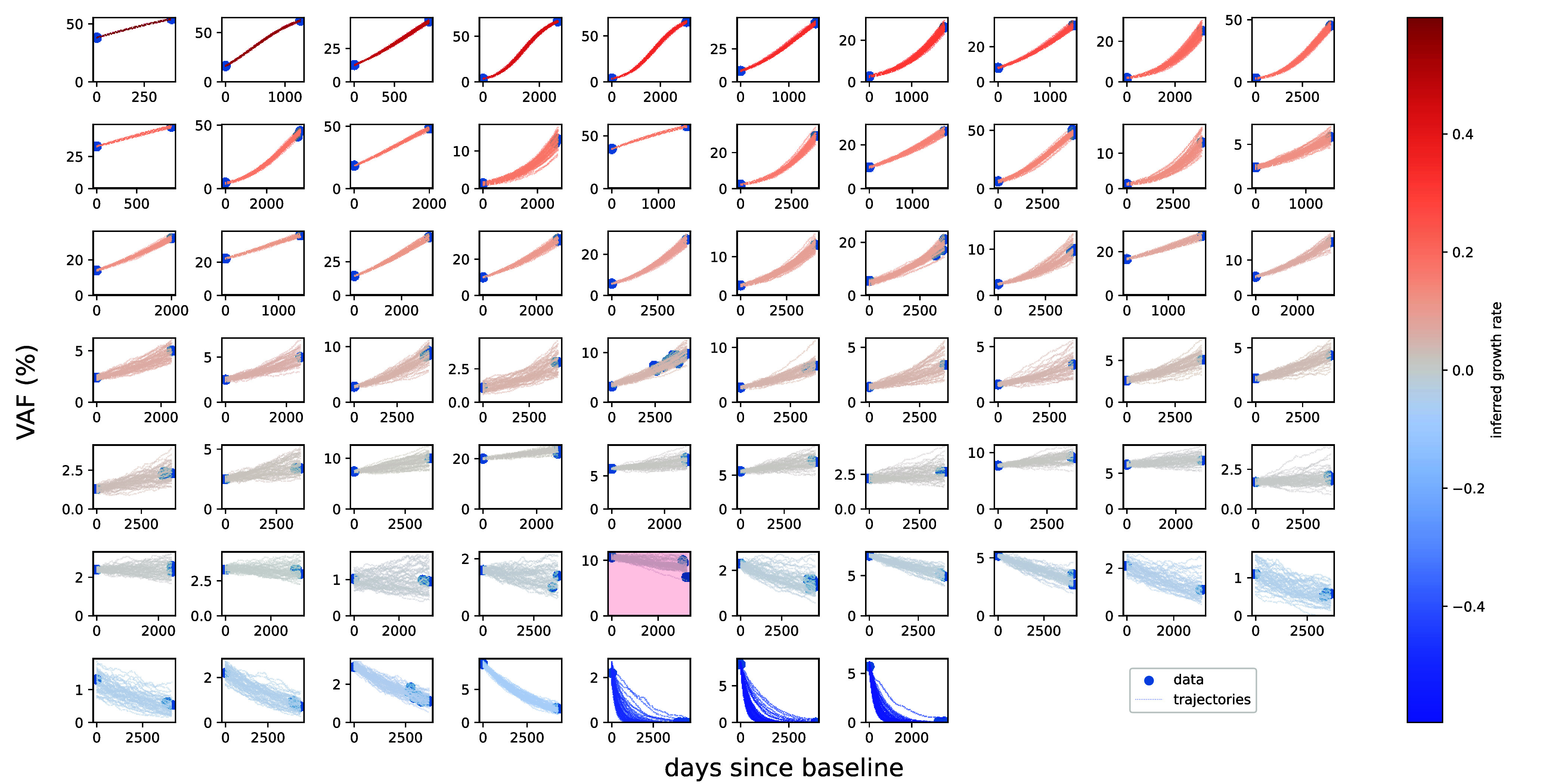
Data (circles) and ensembles of 50 fitted Moran process trajectories for each of the 67 subjects in the >1% *JAK2V617F* GESUS cohort. The horizontal axis is days since the baseline measurement, and vertical axis is the VAF expressed as a percent. For all but one subject (pink subplot), all data points fall within the central 95% interval of the distribution of model trajectories. Here we take $T_{g}=200$ d, and $N=10^{5}$ stem cells in total, and we assume that of *JAK2V617F* mutant stem cells, 50% are homozygous and 50% are heterozygous. Color of trajectories indicates the value of the inferred growth rate. For corresponding results under different assumptions about N, $T_{g}$, and the zygosity ratio, see *SI Appendix*, sections 2.2 and 2.3.

**Fig. 3.**
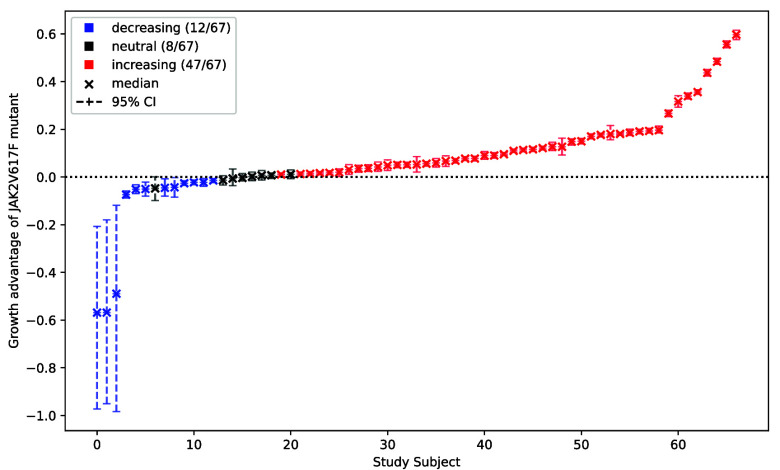
The inferred self-renewal advantage, s, of *JAK2V617F* mutant stem cells over healthy stem cells, with 95% CIs. For each subject, we obtain the distribution of s given the data via Approximate Bayesian Computation. Color indicates whether a given subject’s data indicate statistically significant decline (blue), increase (red), or neither (black), determined by whether s=0 lies above, below, or within the 95% CI, respectively. Here we have assumed $N=10^{5}$. For results corresponding to different choices of N, see *SI Appendix*, section 2.2. Subjects have been sorted according to their median estimated self-renewal advantage.

Fig. 2 depicts data for each of the 67 subjects in the >1% *JAK2V617F* GESUS cohort superimposed with ensembles of trajectories of the fitted Moran process model, and we observe that for 66 of 67 subjects, all data points fall within the central 95% of the distribution of model trajectories. For one subject (subplot highlighted in pink), the first three of their four measurements fall within the central 95% and the fourth does not. Fig. 3 shows estimates with uncertainty of the self-renewal advantage parameter for each individual in our study. We observe that for a substantial fraction (12 of 67) of individuals, the inferred self-renewal is advantage is statistically significantly negative, indicating that their VAF showed a decrease that was too large to have arisen by chance. A majority, as one might have expected, show a significantly positive self-renewal advantage (47 of 67), while the remainder (8 of 67) shows no statistically significant evidence that *JAK2V617F* mutant stem cells have any self-renewal advantage over WT stem cells.

### Additional Sources of Noise.

We now consider the existence of sources of variability in the data that are unexplained by the model. Our model builds in randomness at the level of individual cell self-renewal and death events. However, the data show greater variability over time than a typical model trajectory, and this indicates that there are sources of variability present in real life that are unaccounted for in our model, which we refer to collectively as “biological variation.” Rather than attempt to build such noise sources into the model directly, we performed a systematic comparison between model simulations and the data in order to quantify the variability introduced by all unmodeled sources (for details see *Materials and Methods*). We found that of the postbaseline measurements, roughly 22% (30/134) of them lay statistically significantly outside a distribution of 1,000 model trajectories originating at the previous measured value. For these measurements, the average distance from the 95% CI for the measurement to the central 95% of the model trajectories was 0.6% VAF and the largest such distance was 3.4% VAF.

**Fig. 4.**
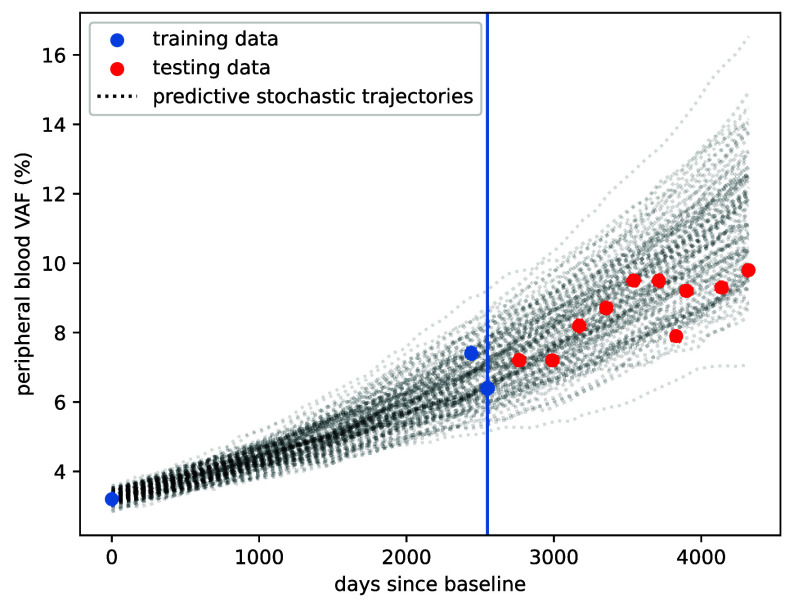
Example of predicting the future evolution of an individual’s VAF using a Moran model fitted to their first three VAF measurements. Circles are data and light dotted lines are stochastic Moran process trajectories based on parameter values sampled by ABC-SMC. The vertical solid line indicates the separation between training (*Left*, blue dots) and testing data (*Right*, red dots). Here we take $T_{g}=200$ d, and $N=10^{5}$.

Next, we address the potential prognostic value of our model. To do this we perform a hypothetical experiment in which, for each subject, we fit the model using only the first three measurements (or the first two in the case that only three measurements were available in total), and attempt to predict the remaining measurements using the fitted model. In Fig. 4 we depict the results of this procedure for the individual from whom we have the most measurements available; corresponding figures for the rest of the 34 subjects for whom we have at least three data points are given in *SI Appendix*, section 4. We find that for 32 of these 34 subjects, all available subsequent measurements lay within the middle 95% of the distribution of model trajectories.

**Fig. 5.**
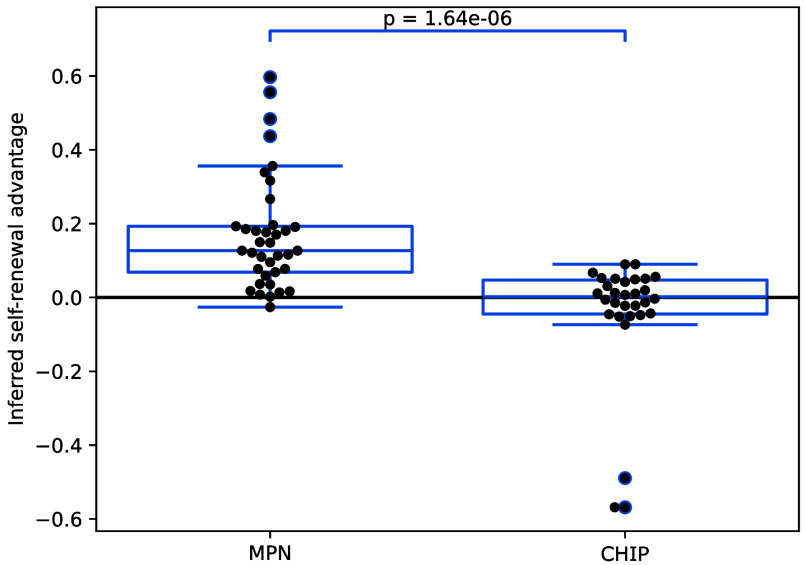
Box plot depicting the distribution of inferred self-renewal advantage among those who were (*Left*, n=37) vs. were not (*Right*, n=30) diagnosed with MPN at the time of this study. While self-renewal advantage is generally higher for those who were diagnosed with MPN, its value has limited predictive ability. Note that there are some individuals who were diagnosed with MPN but who had a negative inferred self-renewal advantage for the *JAK2V617F* clone. Population means are different with significance $p\approx 1.64\times 10^{-6}$ according to a two-sample Welch’s *t* test.

Finally, we comment on the diagnostic value of our model in terms of our ability to predict transition to MPN disease. For each subject, we have information on MPN and related diagnoses as of present. Of the 67 subjects we consider, 37 had received an MPN diagnosis at the time of this study (any of: chronic myeloproliferative syndrome, primary and secondary myelofibrosis, essential thrombocythemia, polycythemia vera, secondary MF after PV, myeloproliferative disease (unspecified), and “MPN”). We group subjects according to whether or not they received an MPN diagnosis and examine the distribution of inferred self-renewal advantage of the mutant clone for each group, see Fig. 5. On average, those who have transitioned to MPN had a significantly higher self-renewal advantage of the *JAK2V617F* clone, but notably, the self-renewal advantage of the *JAK2V617F* clone is not a perfect predictor of disease. In particular, there are individuals for whom we infer a negative self-renewal advantage for the malignant clone, who nonetheless have transitioned to MPN disease. Meanwhile there are also individuals who show significant expansion of the malignant clone who however have not developed MPN disease. This indicates that health factors outside of VAF and its growth rate have a bearing on disease status and progression in MPN. Such health factors may include sources of chronic inflammation (39), incidence of infection or serious injury (40), frequent blood donation, or medications taken for reasons other than hematological malignancy, for example statins to control cholesterol (41, 42).

## Discussion

We have presented data on *JAK2V617F* VAF in 67 individuals from the general population and without treatment for MPN disease, with multiyear follow-up. The data are consistent with a model based on a fixed total number of stem cells where mutated cells experience an advantage in terms of their likelihood to self-replicate compared with normal cells. We have used Approximate Bayesian Computation to infer the value of the self-renewal advantage for each individual. The model fits well for almost all subjects and can reliably predict the future evolution of mutational burden.

We find that for around 70% (47 out of 67) of subjects, mutated cells enjoy a statistically significant advantage over normal cells, while for 18% (12 out of 67), the data correspond to a statistically significant disadvantage of mutated cells relative to normal cells. The final 12% (8 out of 67) do not show statistically significant evidence that the cancer cells are any more or less inclined to self-renew than normal cells. This result stands mostly in contrast to prior studies of *JAK2V617F* mutation, especially those that have been done on MPN patients. This makes sense since studies of MPN patients, by design, include only those individuals in whom the *JAK2V617F* stem cells have become prevalent enough to cause hematological malignancy. By identifying individuals from the general population in whom the *JAK2V617F* VAF stays constant or declines, we create an opportunity to identify other health factors that may predict the expansion or nonexpansion of *JAK2V617F* mutant cells and thereby gain insight into the mechanisms of MPN disease progression.

Further, the fact that several individuals show a significant decline in VAF during the study period indicates that our model, with a fixed self-renewal advantage for the mutant clone, likely does not apply for the entire duration of *JAK2V617F* mutation existing in the person in question. Indeed, if the mutant clone has a disadvantage relative to healthy cells, then it is highly unlikely for the VAF to have risen from a single cell to the significant levels we observe in GESUS participants. This implies that there was a period of time, after the initial mutation but before the decline in VAF, where the *JAK2V617F* mutant cells did enjoy an advantage over healthy cells. The change from advantage to disadvantage may correspond to the development of an adaptive immune response against *JAK2V617F* HSCs.

There are several ways that our modeling work could be changed or extended. First would be to model the process of cell differentiation explicitly. In our model, we make strong assumptions about the differentiation of mutant and wild-type stem and progenitor cells, the proportion of homo- vs. heterozygous cells, as well as the impact of homo- vs. heterozygosity on cell behavior (38). These assumptions are difficult if not impossible to validate given only data from peripheral blood. It is likely that our assumptions are not fully correct, and including cell differentiation into our model, especially in a way that depends on mutation status and zygosity, would make our model more realistic and potentially more accurate. Ideally we would calibrate a differentiation model using simultaneous cell count measurements at different differentiation stages (i.e. stem/progenitor and mature), which has been done previously (37).

Another important future direction is, as alluded to above, to search for predictors of VAF growth rate in other health data. There is a growing body of literature to indicate that chronic inflammation may be a driving factor in cancer progression generally, and specifically in CHIP (43) and MPN (44–51). To investigate these issues in the >1% *JAK2V617F* GESUS cohort, we have measurements of complete blood cell counts including hematocrit and hemoglobin, as well as C-reactive protein (CRP). Statistics of these quantities in the CH and MPN subgroups of the >1% *JAK2V617F* GESUS cohort are given in Table 1. Age, sex, smoking status, and BMI do not show statistically significant differences between CH and MPN, while most blood cell counts do, as does the composite variable indicating any elevated blood cell count. A more thorough analysis that also includes GESUS participants with <1% *JAK2V617F* VAF at baseline can hopefully identify criteria that distinguish those whose VAFs increase from those whose VAFs do not increase. Such results would have importance not only for early detection and prognosis, but potentially also for prevention. A preliminary correlation analysis of inferred growth rate with selected clinical variables is presented in *SI Appendix*, section 5.

**Table 1.** Baseline characteristics by later MPN progression

	CH N (%) / Mean (SD)	MPN progression N (%) / Mean (SD)	*P*-value
Number of individuals	30 (44.8)	37 (55.2)	
Sex			
Female	10 (33.3)	21 (56.8)	*0.09*
Male	20 (66.7)	16 (43.2)	
Age	59.8 (10.4)	59.2 (11.7)	*0.8*
Smoking			
Never smoker	13 (43.3)	12 (32.4)	*0.6*
Former smoker	10 (33.3)	16 (43.2)	
Current smoker	7 (23.3)	9 (24.3)	
BMI (kg/m2)	25.8 (3.2)	25.7 (3.9)	*0.9*
Laboratory test			
VAF (%)	3.5 (2.6)	9.6 (9.8)	*0.0008*
Leukocytes ($\times 10^{9}$/L)	6.8 (1.1)	8.8 (2.2)	*1.6x10-5*
Neutrophils ($\times 10^{9}$/L)	3.9 (0.7)	5.4 (1.7)	*1.3x10-5*
Eosinophils ($\times 10^{9}$/L)	0.2 (0.1)	0.3 (0.1)	*0.02*
Basophils ($\times 10^{9}$/L)	0.04 (0.02)	0.07 (0.06)	*0.007*
Lymphocytes ($\times 10^{9}$/L)	2.2 (0.8)	2.4 (0.8)	*0.3*
Monocytes ($\times 10^{9}$/L)*	0.52 (1.3)	0.60 (1.3)	*0.03*
Thrombocytes ($\times 10^{9}$/L)	296 (75.8)	400 (164)	*0.001*
Erythrocytes ($\times 10^{12}$/L)	4.7 (0.4)	5.0 (0.4)	*0.01*
Hemoglobin (mmol/L)	8.8 (0.7)	9.2 (0.8)	*0.03*
Hematocrit (ratio)	0.43 (0.03)	0.46 (0.04)	*0.006*
hsCRP (mg/L)*	1.15 (2.2)	1.25 (3.5)	*0.7*
Elevated blood cells			
No elevated blood cells	20 (69.0)	8 (21.6)	*0.0003*
Elevated blood cells	9 (31)	29 (78.4)	

Abbreviations: CH: Clonal hematopoiesis, MPN: Myeloproliferative neoplasms.

^*^Calculated geometric mean and SD for monocyte count and hsCRP.

A composite variable for elevated blood cell counts was made if more than one myeloid or lymphoid cell lineage was above the normal range, as defined by laboratory reference values and sex: hemoglobin concentration >10.5 mmol/L (male) or >9.5 mmol/L (female), hematocrit >0.50 (male) or >0.46 (female), erythrocytes >$5.7\times 10^{12}$/L (male) or >$5.2\times 10^{12}$/L (female), thrombocytes >$390\times 10^{9}$/L, leukocytes >$8.8\times 10^{9}$/L, neutrophils >$7.0\times 10^{9}$/L, monocytes >$0.7\times 10^{9}$/L, eosinophils >$0.5\times 10^{9}$/L, basophils >$0.1\times 10^{9}$/L, and lymphocytes >$3.5\times 10^{9}$/L.

## Materials and Methods

### Data.

We use data from the Danish General Suburban Population Study (GESUS), which had an inclusion period from 2010 to 2013 in Næstved Municipality, Denmark (22, 24). The study was approved by a scientific, ethical committee and the Danish Data Protection Agency. Participants provided written informed consent. The study complied with the principles of the Helsinki Declaration. In connection with GESUS, 19,958 individuals were screened for MPN-associated mutations. Of these, 645 were found to be mutation positive [either *JAK2V617F* (613) or *CALR* (32)]. After removing 16 individuals who were found to have MPN disease at the time of baseline measurement, there were 629 individuals with CHIP at baseline, of whom 599 had *JAK2V617F* and 30 had *CALR* mutation. The 599 individuals with *JAK2V617F* CHIP were further stratified by allele burden, *JAK2V617F*≥ 1% (92) or *JAK2V617F*<1% (507).

**Fig. 6.**
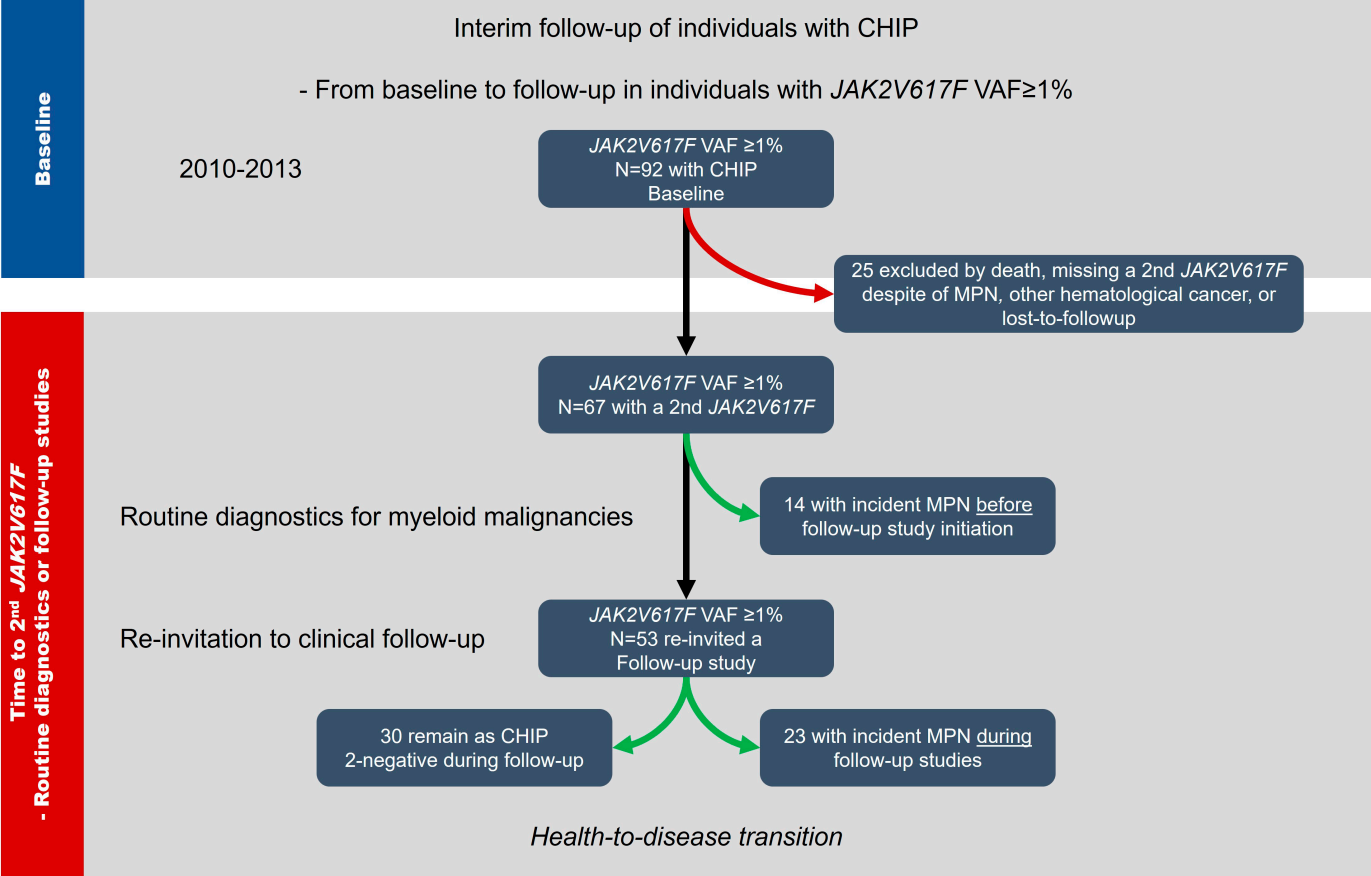
Flowchart depicting the selection of the >1% *JAK2V617F* GESUS cohort from the Danish General Suburban Population Study (GESUS).

**Fig. 7.**
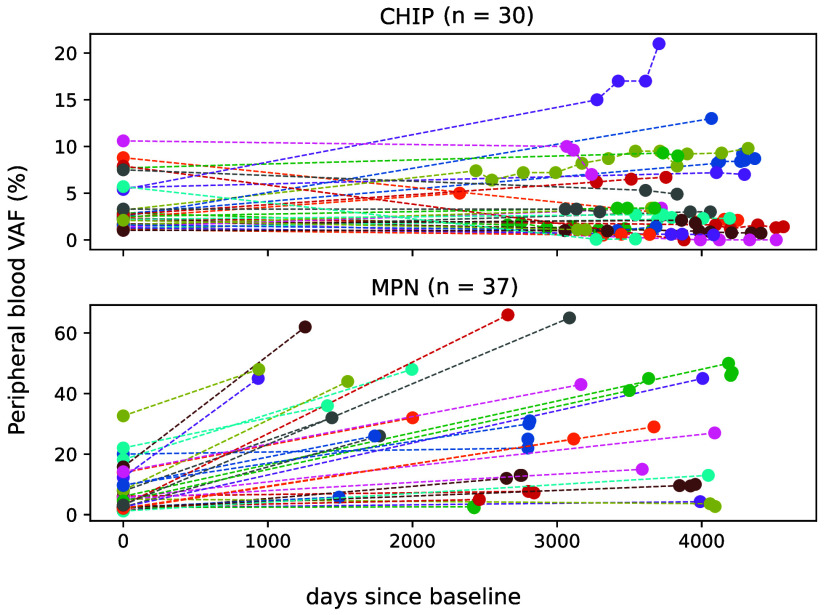
Overview of VAF over time for GESUS participants with *JAK2V617F*≥ 1% at baseline and at least two measurements. Each color corresponds to one individual (n=67) and all data shown here were taken in the absence of any treatment for MPN disease. The *Upper* panel is from subjects without any MPN diagnosis at time of analysis, and *Lower* panel is from subjects who eventually received an MPN diagnosis after baseline measurement and before time of analysis.

Of the 92 individuals with *JAK2V617F*≥ 1% CHIP at baseline, 25 were ineligible for follow-up due to one or more of: death; missing a second *JAK2V617F* measurement despite MPN diagnosis; other hematological malignancy; or lost-to-follow-up. Sixty-seven individuals were identified with a second JAK2V617F measurement during follow-up of which, 53 were between 2020 and 2024, and 14 prior to 2020 (see flowchart in Fig. 6). These 67 individuals comprise the “>1% *JAK2V617F* GESUS cohort” of the present study. We have between 2 and 13 *JAK2V617F* VAF measurements for each member of the >1% *JAK2V617F* GESUS cohort. The data are presented in Fig. 7. Note that VAF at follow-up is not strictly determined by VAF at baseline, i.e. there are some individuals showing an increase from a low level and others showing nonincrease or even decrease from a moderate or high level. This indicates that the *JAK2V617F* mutation expands differently in different people, and it is our goal to quantify and attempt to account for this heterogeneity.

Of the 67 individuals in the >1% *JAK2V617F* GESUS cohort, 37 were diagnosed with MPN disease at some point after the baseline measurement, while the remaining 30 remained classified as CHIP (Clonal Hematopoiesis of Indeterminate Potential) (52). A table of summary statistics of blood cell counts and hsCRP for CHIP vs. MPN progression groups is shown in Table 1.

### Moran Process.

Our goal is to formulate a mathematical model describing the *JAK2V617F* VAF in a given individual as a function of time. As *JAK2V617F* is known to originate in the hematopoietic stem cells (HSCs), we formulate our model at the level of stem cells, and suppose that the VAF we measure in peripheral blood (i.e. among mature nucleated blood cells) is reflective of the clonal makeup of the stem cell compartment.

We would like our model to be “as simple as possible but no simpler,” which means it should reflect a reasonable facsimile of what we believe to be the most important biological features underlying the dynamics of *JAK2V617F* VAF, while allowing for inference of biologically meaningful parameters from limited data. For this purpose we formulate a simple model consisting of a single integer variable that evolves in time, specified by a single free parameter.

The assumptions that go into our model are as follows:

1. The number of stem cells, N, does not change with time.
2. The mutational status of any given stem cell can be characterized as wild type (WT) or mutant (M), by which we mean harboring the *JAK2V617F* mutation.
3. The mutational status of any given stem cell affects its propensity to self-replicate by division.
4. Stem cells divide at a fixed rate, corresponding to each cell dividing on average once every $T_{g}=200$ d. Estimates for the typical time between divisions of hematopoietic stem cells range from once in 23 wk (161 d) to once in 67 wk (469 d) (34, 35). See *SI Appendix* for a discussion of the impact of choice of $T_{g}$.
5. Stem cells die at exactly that rate that keeps the total population size fixed, and mutational status does not affect a cell’s propensity to die.

These assumptions are quite strong, but they have an advantage that they result in a simple mathematical description with one free parameter, the relative propensity for cell division of mutant vs. wild-type cells, that has biological meaning and can be assessed on a per-individual basis given longitudinal data on VAF. The mathematical model obeying these assumptions is known as a Moran process (33), and it, as well as the closely related Wright–Fisher model (53), has been used extensively to model various biological systems including disorders driven by HSCs (43, 54–67).

Mathematically, our model can be defined as a stochastic (i.e. random) process that takes place in discrete time steps. The real-world time corresponding to one time step in the model is chosen so that on average, each stem cell divides once each $T_{g}$ days. In other words, the total number of time steps (i.e. cell divisions) in $T_{g}$ days should be equal to N, the total number of stem cells. This means that one model time step is equivalent to $T_{g}/N$ days. We denote by X(t) the number of stem cells harboring the *JAK2V617F* mutation at the $t^{\mathrm{th}}$ time step. To progress to the next time step, one cell must divide and one cell must die. The cell that is to divide is chosen at random from among the entire collection of cells, with WT cells selected with a weight of 1 and M cells selected with a weight 1+s, where s is the single free parameter of our model. The cell that is to die is selected uniformly at random, irrespective of mutational status.

The transition probabilities $P_{i,j}P\left[X\left(t+1\right)=j|X\left(t\right)=i\right]$ are given by

$$\begin{array}{cc} P_{0,0} & =1\\ P_{i,i-1} & =\frac{N-i}{(1+s)\;\cdot \;i+N-i}\cdot \frac{i}{N}\\ P_{i,i} & =1-P_{i,i-1}-P_{i,i+1}\\ P_{i,i+1} & =\frac{(1+s)\;\cdot \;i}{(1+s)\;\cdot \;i+N-i}\cdot \frac{N-i}{N}\\ P_{N,N} & =1 \end{array}$$

Given these transition probabilities it is possible to simulate random trajectories starting from any given initial condition. Some examples of trajectories generated in this way are shown in Figs. 1 (*Right*) and 8. The central aim of our work is to infer, for each individual in the study, a value for the selective advantage parameter s that reproduces their history of VAF over time.

**Fig. 8.**
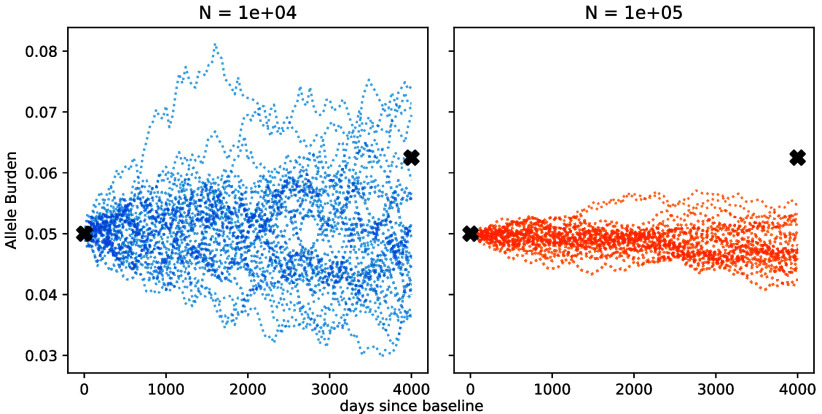
Ensembles of Moran model trajectories with different values of N, showing that the variance after a given number of days is different if we assume $N=10^{4}$ vs. $N=10^{5}$. The number of model time steps is taken to be $t=TN/T_{g}$ where T=4,000 is the total number of days and $T_{g}=200$ is the typical generation time of one stem cell (in days). Black X’s are hypothetical data, corresponding to a VAF of 5% at baseline and 6.25% at follow-up. By visual inspection, these data are consistent with neutral drift if we assume $N=10^{4}$, but inconsistent with neutral drift if we assume $N=10^{5}$.

The most basic question we can ask is whether or not the *JAK2V617F* mutant stem cells have a significantly different self-renewal behavior than WT cells. In mathematical terms, this means asking whether the parameter s is significantly different from 0. Equivalently, we can ask whether or not the observed change in VAF from one measurement to the next is statistically consistent with the Moran process with s=0. In practice, we assess this by checking whether s=0 falls within a specified quantile of the distribution of samples of s generated by the ABC SMC procedure outlined below. Importantly, the answer to this question depends on what we assume to be the total number of stem cells, N. For an illustration of why this is, see Fig. 8, and for a more detailed discussion see *SI Appendix*.

### Estimating the Biological Variation.

Here we describe our approach for estimating the size of noise sources present in the data but absent in our model. For each subject, we have some number of measurements over time (between 2 and 13), with the first measurement serving as the baseline measurement. In order to quantify the amount of unmodeled noise in the data, we did the following for each subject in the GESUS CHIP cohort:

- Simulate an ensemble of 1,000 model trajectories originating at the baseline measurement value, using as the growth rate the median of the posterior distribution obtained by ABC-SMC inference.
- Compare the ensemble of simulated trajectories to the next available measurement
  - If the 95% CI of the measurement overlaps with that of the ensemble of simulations, record zero discrepancy.
  - If 95% intervals do not overlap, record the distance between the intervals as the discrepancy. The units of this distance are VAF percentage points (% VAF).
- Repeat the procedure, using the next available measurement as the baseline and simulating until the following measurement, until all postbaseline measurements have been used.

**Fig. 9.**
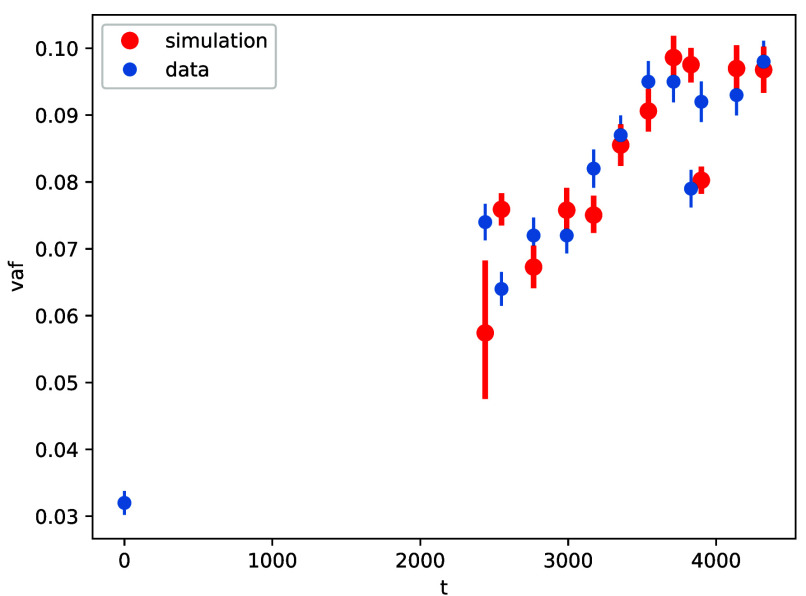
Visualization of the procedure described above for comparing variability in the data to that accounted for by the stochastic model. Blue dots are VAF measurements over time for a single cohort member, with lines representing an estimated 95% CI based on data concerning the reproducibility of ddPCR (68). Red dots and lines indicate 95% intervals for the ensembles of trajectories initiated at the previous measured VAF value.

The result can be visualized in a figure as in Fig. 9. Error bars for measurements represent analytical variation are based on a formula $\sigma \approx \sqrt{\mu }/200$, where σ is the SD and μ is the mean, which was obtained from data on the reproducibility of the ddPCR assay used in this study that were previously published in ref. 68, see *SI Appendix*, section 1 for details.

### Approximate Bayesian Computation.

Here we briefly describe the method of Approximate Bayesian Computation (ABC) (36), a method for sampling from the posterior distribution of model parameters (here the mutant selective advantage, s, and the initial number of mutated stem cells, $N_{0}$) given data (here measurements of allele burden over time, $\left\{\left(t_{1},y_{1}\right),\cdots ,\left(t_{n},y_{n}\right)\right\}$), a prior distribution over model parameters, $p_{0}\left(s,N_{0}\right)$, and a desired total number of samples from the posterior, m.

As a preprocessing step, we convert the measured allele burdens $\left\{y_{j}\right\}$ in peripheral blood to equivalent clonal fractions in the stem cell compartment. We do this by solving for X(t) in Eq. **1**, which yields:

[2] $$\begin{array}{c} X\left(t\right)=\frac{\text{VAF}(t)}{\left(1-k\right)\;\text{VAF}\left(t\right)+\left(\frac{1}{2}kz+k\left(1-z\right)\right)} \end{array}$$

We denote the resulting clonal fractions $c_{j}$.

We first describe the simplest version of the algorithm, sometimes referred to as rejection ABC, which proceeds as follows:

1. While the number of accepted samples is less than the desired total, m, do:
  (a) Sample candidate values of s and $N_{0}$ from the prior distribution $p_{0}\left(s,N_{0}\right)$ (we take a uniform distribution on (−1,0.7) for s and a uniform distribution from 0 to N for $N_{0}$)
  (b) Simulate a trajectory X(t) using the candidate value of s and the initial condition $X\left(0\right)=N_{0}$
  (c) Compute the mean-square difference between the simulated trajectory and the data: d=1n∑j=1n(X(tj)−cj)2
  (d) If d<ϵ, then accept the candidate values of s and $N_{0}$

The threshold parameter ϵ should be taken as close to zero as possible while still accepting the desired number of samples within a reasonable amount of computing time. If it is not possible to reduce ϵ to a value indicating good agreement between the simulations and the data, then this is evidence that the model (including the prior distribution $p_{0}$) does not accurately reflect the data and should be changed.

We employ a modified version of ABC known as ABC SMC (Sequential Monte Carlo), as implemented in the ApproxBayes.jl package in Julia (69) The basic idea of ABC SMC is to apply rejection ABC with a large value of ϵ, then use the resulting posterior distribution as the prior for another round of rejection ABC with a smaller value of ϵ, taking care to set the acceptance probability appropriately. This procedure is repeated until a termination condition is reached, typically defined by a desired value of ϵ, which we set to $10^{-3}$. We obtain m=1,000 samples for most cases, but reduce this to m=200 for more compute-intensive runs (i.e. those with larger N). Further details can be found in ref. 36 and at https://github.com/marcjwilliams1/ApproxBayes.jl.

## Supplementary Material

Appendix 01 (PDF)

## Data Availability

Some study data are available. Code used for the analysis in this paper can be found at https://doi.org/10.5281/zenodo.19922836 (70) and https://doi.org/10.5281/zenodo.19922873 (71). Data cannot be shared publicly due to the following reasons: the European Union’s General Data Protection Regulation, the fact that the participants/patients did not consent to the publication of their raw data; and last, the scientific ethical committee did not approve the publication of participants’/patients’ raw data. To request access to data, contact Christina Ellervik (cel@regionsjaelland.dk) or Hans Hasselbach (hkhl@regionsjaelland.dk).
