# Supplementary material for: Mathematical modeling of JAK2V617F clonal expansion in a general population cohort
Source: Proc Natl Acad Sci U S A. 2026 Jun 17;123(25):e2507773123. doi: 10.1073/pnas.2507773123 (PMC13291664; doi:10.1073/pnas.2507773123)
Supplement: Supplementary file 1 — Appendix 01 (PDF) [file pnas.2507773123.sapp.pdf]

SI Appendix to article: Mathematical modeling of JAK2V617F clonal expansion in a general population cohort

Jordan Snyder[a,1], Morten Andersen[a], Johanne Gudmand-Høyer[a], Morten Kranker Larsen[b], Vibe Skov[b], Lasse Kjær[b], Christina Schjellerup Eickhardt-Dalbøge[b], Trine A. Knudsen[b], Christina Ellervik[d,e,f,g], Hans C. Hasselbalch[b], Johnny T. Ottesen[a,*], and Thomas Stiehl[a,c,*]

[a]Centre for Mathematical Modeling - Human Health and Disease, IMFUFA, Department of Science and Environment, Roskilde University, Roskilde, Denmark
[b]Department of Hematology, Zealand University Hospital, Roskilde, Denmark
[c]Institute for Computational Biomedicine - Disease Modeling, RWTH Aachen University, Aachen, Germany
[d]Department of Clinical Medicine, Faculty of Health and Medical Sciences, University of Copenhagen, Copenhagen, Denmark
[e]Department of Laboratory Medicine, Boston Children's Hospital, Boston, MA, USA
[f]Department of Pathology, Harvard Medical School, Boston, MA, USA
[g]Department of Clinical Biochemistry, Zealand University Hospital, Køge, Denmark
[*]These authors contributed equally as last author

May 1, 2026

1 Estimating error bars for VAF measurements

To estimate uncertainties on the VAF measurements used in this study, we refer to previously reported data for the ddPCR assay used in this study [3]. Supplementary Table 1 of [3] reports five replicate measurements of 40 different samples across a range of VAF values. For each of the 40 samples, the authors

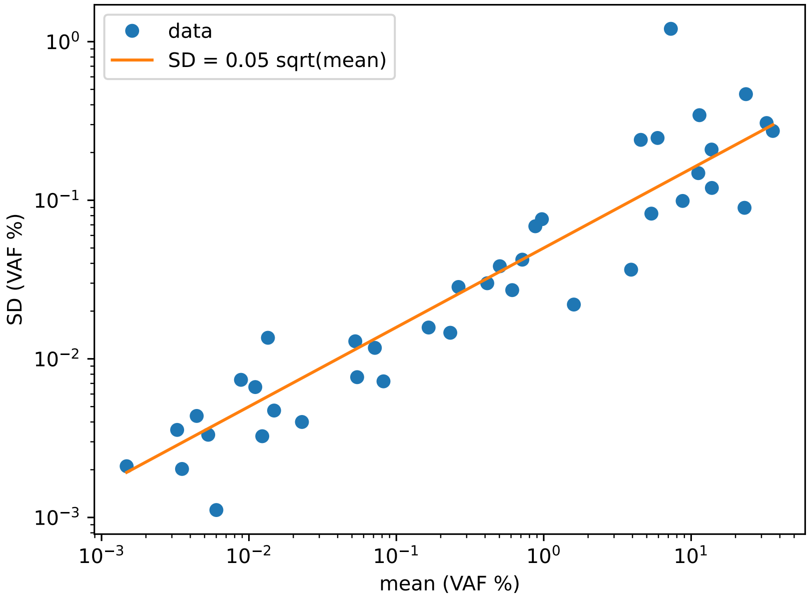

Figure S1: Scatter plot in log-log scale of the data reported by Kristiansen et al. [3] regarding the reproducibility of the ddPCR assay used in the GESUS study. The solid line is a fitted function $\Sigma = 0.05\sqrt{M}$. Note that the units are percent VAF, i.e. they range from 0 to 100.

obtain thus a mean M and a standard deviation Σ, measured in percentage points (0-100). Plotting these 40 (M, Σ) pairs on a log-log scale nicely reveals an approximate power law relationship, with slope (exponent) very close to 1/2. The figure S1 shows a good fit to the function $\Sigma = \sqrt{M}/20$. Since we work mostly with VAF as a fraction, we are interested in $\mu = M/100$ and $\sigma = \Sigma/100$. Changing variables to μ, σ, we get

$$\Sigma \approx \frac{1}{20}\sqrt{M} \tag{1}$$

$$100\sigma \approx \frac{1}{20}\sqrt{100\mu} \tag{2}$$

$$\sigma \approx \frac{1}{2000}10\sqrt{\mu} \tag{3}$$

$$\sigma \approx \frac{1}{200}\sqrt{\mu} \tag{4}$$

which we use in the main text.

2 Robustness to hyperparameter selection

2.1 General mathematical considerations for stem cell number

The total number of stem cells, N, is an important free parameter in the Moran process model, in particular because it affects the variability present in the model. The choice of N therefore influences whether or not a given set of observations are consistent with the Moran model with a given value of the drift s. To address this issue precisely, we can make use of the following formula for the conditional variance of the Moran process with neutral drift (i.e. $s = 0$):

$$\mathrm{Var}(X(t) \mid X(0) = i) = \tfrac{2i}{N}\left(1 - \tfrac{i}{N}\right) \frac{1 - \left(1 - \frac{2}{N^2}\right)^t}{\frac{2}{N^2}} \tag{5}$$

where t is the number of time steps of the Moran process. Since we measure VAF rather than absolute counts of mutant cells, we convert the above expression for variance of the number of mutant cells into one for the variance of the fraction of mutant cells, $p(t) := X(t)/N$. Simplifying, we have

$$\mathrm{Var}(p(t) \mid p(0) = p_0) = p_0\,(1 - p_0)\left[1 - \left(1 - \frac{2}{N^2}\right)^t\right] \tag{6}$$

As mentioned previously, the number of model time steps corresponding to a given number of days depends on the total number of cells, N, and the average time between cell divisions for each cell, T_g. In particular, a real life time span of T days is equivalent to $t = TN/T_g$ time steps. Inserting this expression into Eq. (6) gives

$$\mathrm{Var}(p(t = TN/T_g) \mid p(0) = p_0) = p_0\,(1 - p_0)\left[1 - \left(1 - \frac{2}{N^2}\right)^{\frac{NT}{T_g}}\right] \tag{7}$$

$$\approx p_0\,(1 - p_0)\left[1 - \exp\left(\frac{-2T}{NT_g}\right)\right]. \tag{8}$$

In the second line we have approximated the power term by an exponential to make it easier to read. For values of N, T, T_g relevant here, the two expressions are in very good agreement. We also note that for large N, the right-hand side of Eq. (6) scales like $1/N$ (see Fig. S2). This is an important observation because it highlights how the value we choose for N affects our determination of how large a change in VAF can be explained by neutral drift.

To see how this issue affects our determination of whether or not a given individual's VAF measurements are consistent with neutral drift, we have performed a parameter sweep over N and checked, for each possible value of N, whether or not each individual's VAF measurements are consistent with neutral drift. The results are collected in Fig. S3. The sweep was taken for N values

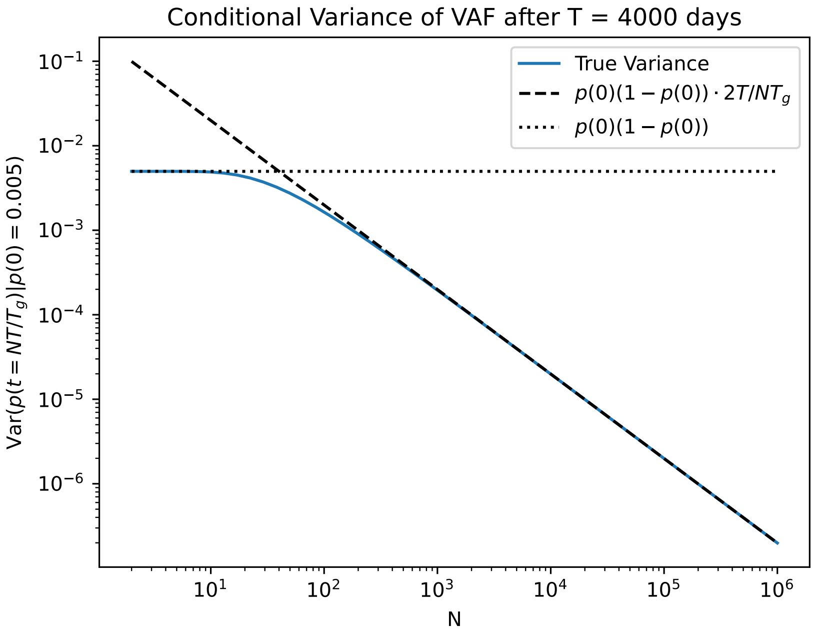

Figure S2: Overview of the variance in VAF as predicted by the Moran model where the mutant clone has no selective advantage (i.e. neutral drift). Note that for large N, the variance scales like $1/N$, while for small N, it approaches a limit value of $p(0)(1-p(0))$. This is because when N is small compared to T/T_g, almost all trajectories reach absorption at $p = 0$ (with probability $p(0)$ or $p = 1$ (with probability $1 - p(0)$). True variance is computed using the formula Eq. (7).

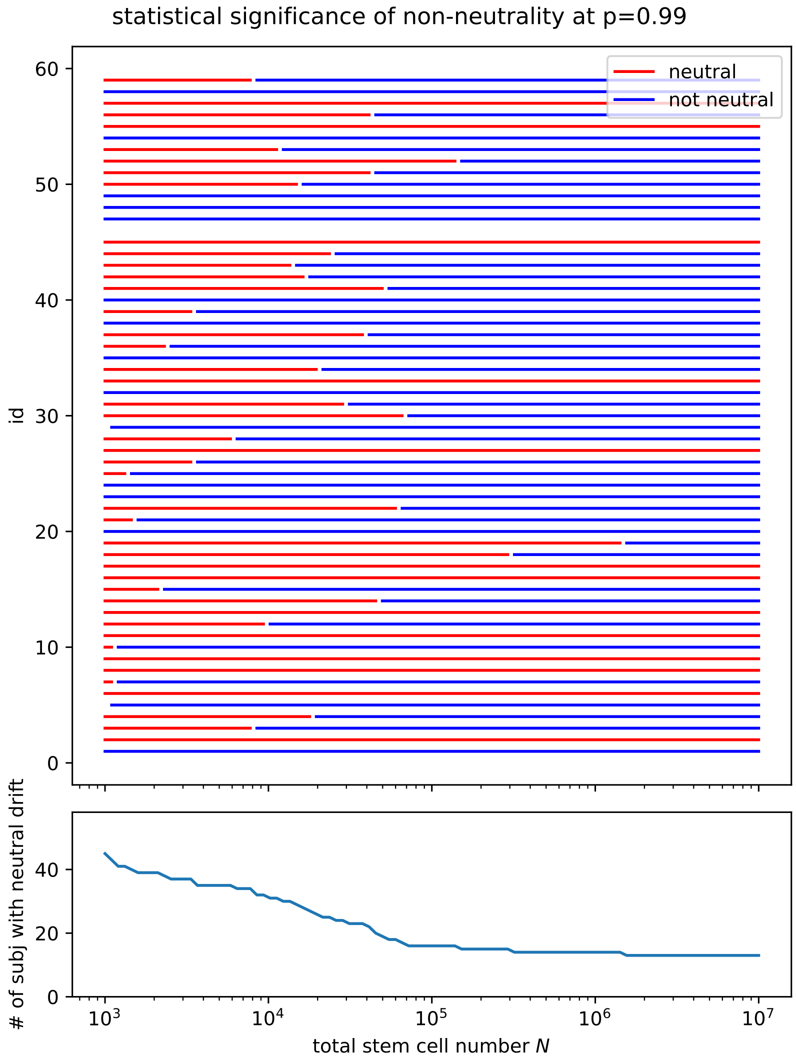

Figure S3: A visual summary of whether or not, for each individual, their data are consistent with neutral drift under the Moran process model. Each horizontal line corresponds to a person, and the line color indicates whether (red) or not (blue) the individual's VAF measurements are consistent with neutral drift at a given N value. Individuals are ordered by GESUS cohort ID number. Lower plot shows the total number of individuals determined to be consistent with neutral drift at a given N value. Note that as N increases, the variance in the model decreases, which causes fewer individuals to be judged consistent with neutral drift.

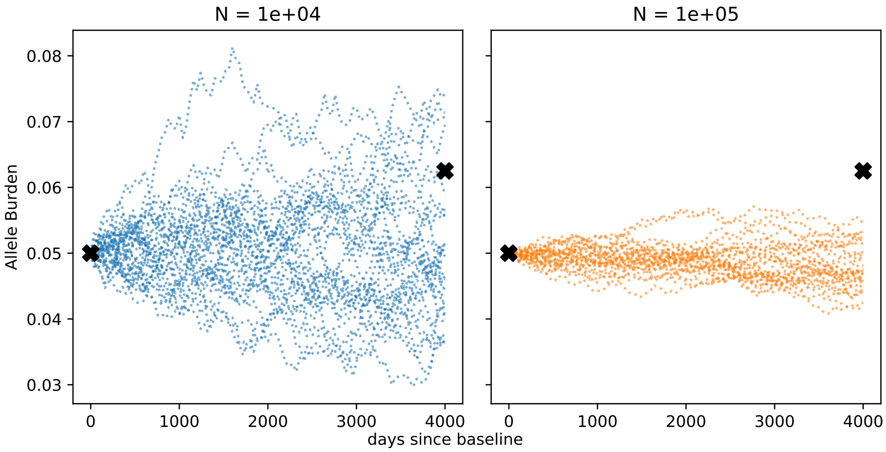

Figure S4: Ensembles of Moran model trajectories with different values of N, showing that the variance after a given number of days is different if we assume $N = 10^4$ vs $N = 10^5$. The number of model time steps is taken to be $t = TN/T_g$ where $T = 4000$ is the total number of days and $T_g = 200$ is the typical generation time of one stem cell (in days). Black X's are hypothetical data, corresponding to a VAF of 5% at baseline and 6.25% at follow-up. By visual inspection, these data are consistent with neutral drift if we assume $N = 10^4$, but inconsistent with neutral drift if we assume $N = 10^5$.

ranging from 10^3 to 10^7, which comfortably contains all reasonable estimates of the size of the stem cell compartment (which are generally between 10^4-10^5).

The choice of N also affects how consistent our data are with the Moran process model in the case when s is different from zero. In such cases, we can generate an ensemble of model trajectories based on the value of s inferred by ABC SMC and compare that ensemble to data, as in Fig. S4. For different choices of N, the distribution of model trajectories will be different, and in particular for larger N the distribution will be more narrow. This leads to the phenomenon that for large enough N, the data in fact lie outside the distribution of the stochastic model trajectories corresponding to the best-fit value of s. To see the difference compare Fig. S5 to Fig. S6. If the inherent variability of the Moran process were the only source of noise in this system, then this would provide an upper bound on the number of HSC. However, there almost certainly is considerable variability due to many factors, including blood cell differentiation, other disease or health factors, and sampling and assay uncertainty.

2.2 Robustness of inference results to stem cell number and cycling rate

Next we discuss robustness of the distribution of growth rate s under the Moran process model, as inferred using ABCSMC, to different choices of the total stem

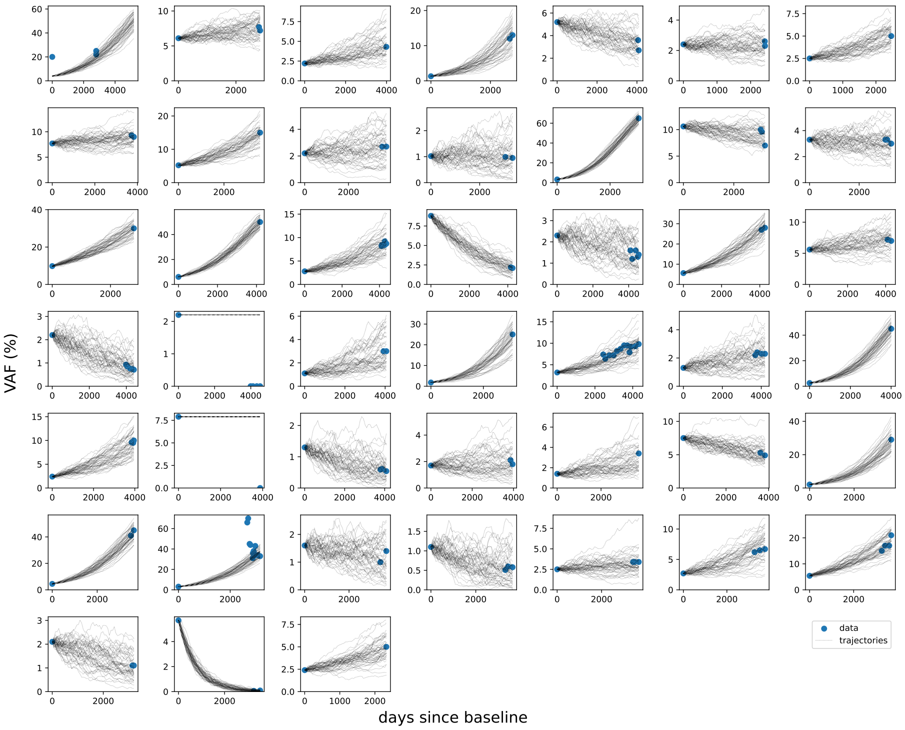

Figure S5: Comparison of data to ensembles of Moran process trajectories using the value of s inferred by linear regression to an approximate ODE model, assuming $N = 10^4$. Each panel corresponds to one study subject, and individuals are ordered by GESUS cohort ID number.

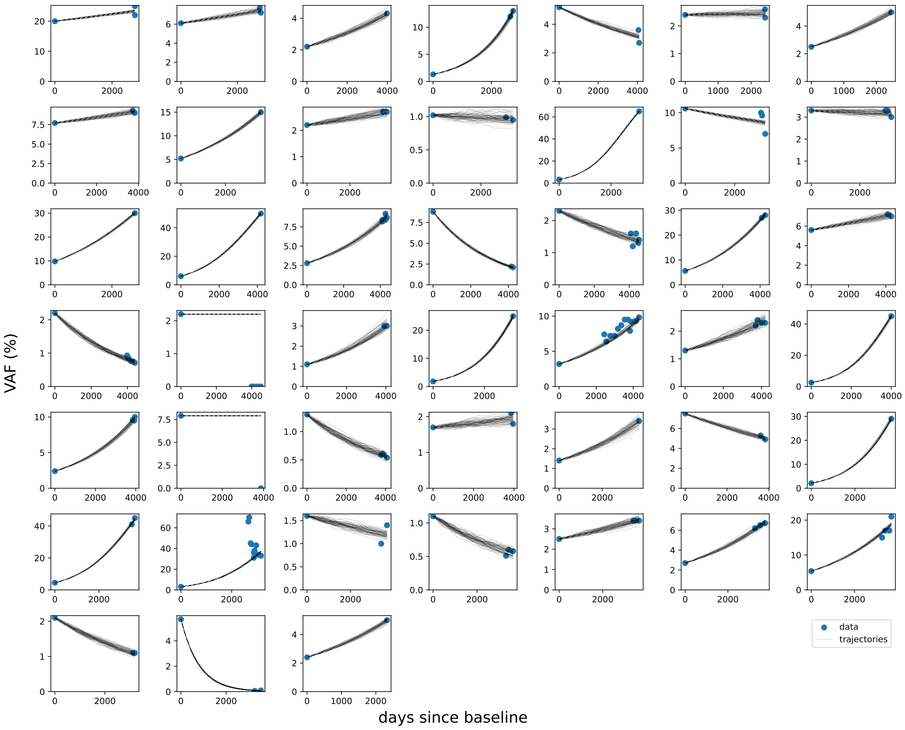

Figure S6: Comparison of data to ensembles of Moran process trajectories using the value of s inferred by linear regression to an approximate ODE model, assuming $N = 10^6$. Each panel corresponds to one study subject, and individuals are ordered by GESUS cohort ID number.

cell number N and the characteristic time of stem cell division, T_g. Estimates for N range from 10^4 to 2×10^5, while estimates for T_g range from 23 weeks (161 days) to 67 weeks (469 days). The main text reports results for $N = 10^5$ and $T_g = 200$ days. Here we report complementary results for $N \in \{10^4, 5 \times 10^4, 10^5, 2 \times 10^5\}$ and $T_g \in \{100, 200\}$. Due to limitations on computation time, we were unable to run the inference procedure to convergence inference for all subjects for all parameter values.

The key quantity of interest being inferred is the self-renewal advantage of mutant cells, denoted throughout by s. Below, in Figures S7–S73 we present box-and-whisker plots of the inferred distribution of s for each subject, as a function of N and for both values of T_g (100 and 200 days)

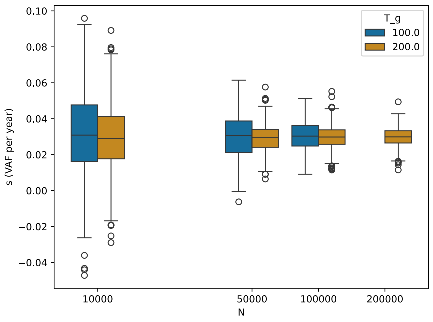

Figure S7: Subject 1

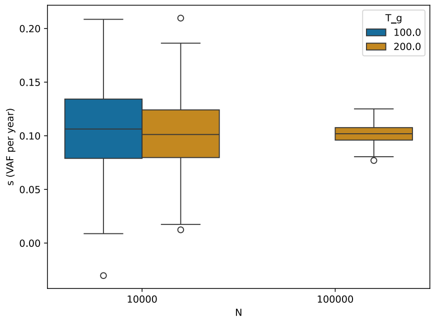

Figure S10: Subject 6

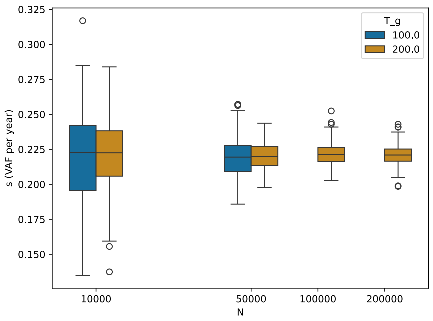

Figure S8: Subject 4

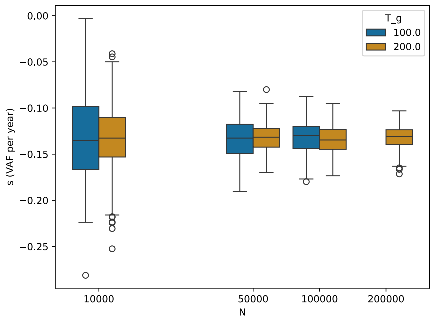

Figure S11: Subject 8

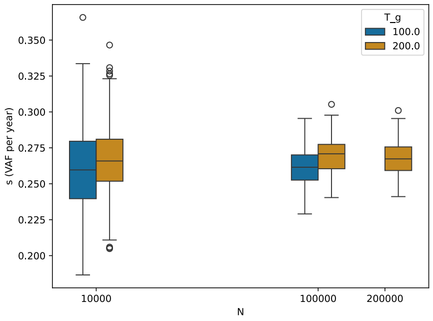

Figure S9: Subject 5

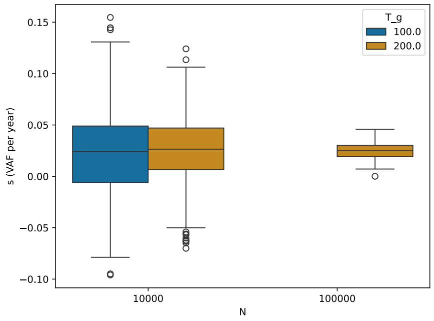

Figure S12: Subject 10

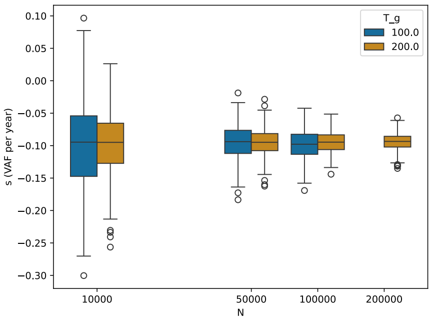

Figure S13: Subject 12

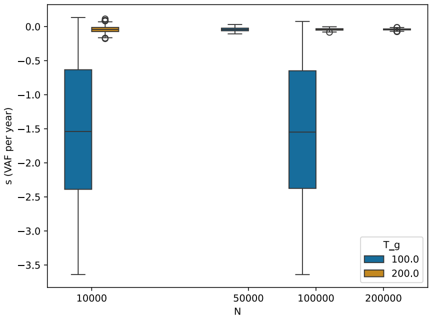

Figure S14: Subject 13

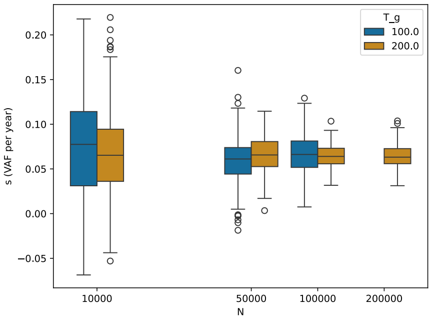

Figure S15: Subject 14

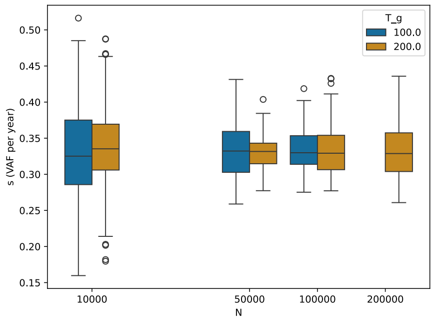

Figure S16: Subject 15

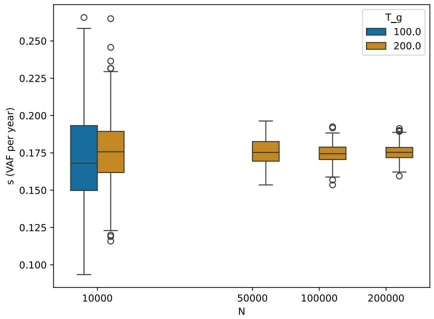

Figure S17: Subject 16

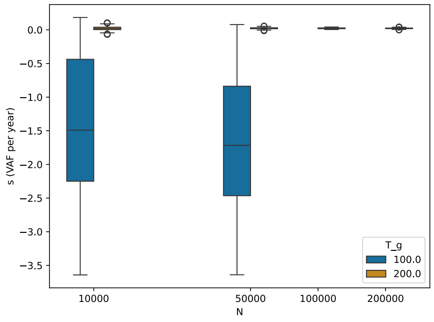

Figure S18: Subject 17

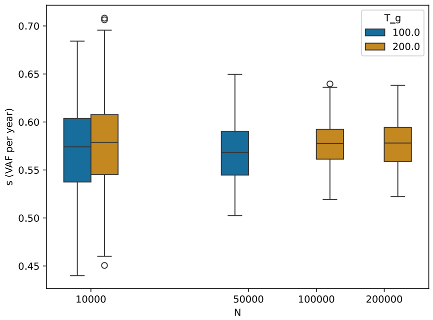

Figure S19: Subject 20

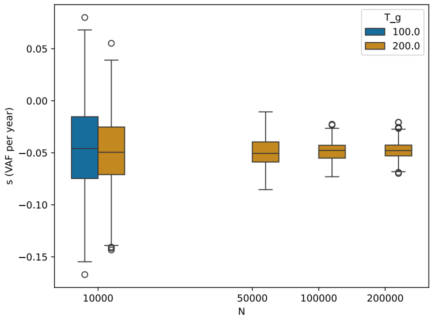

Figure S20: Subject 21

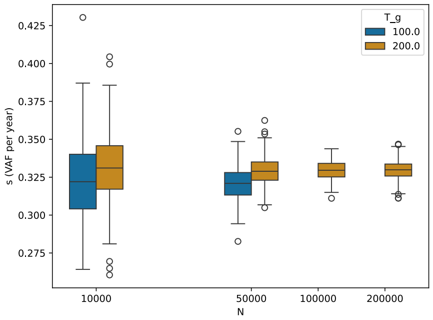

Figure S21: Subject 22

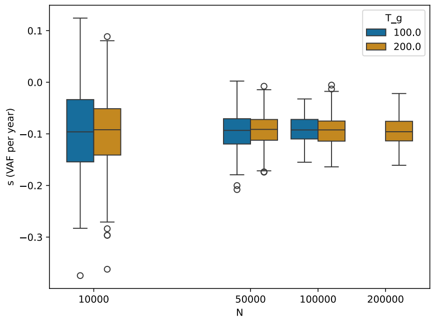

Figure S22: Subject 25

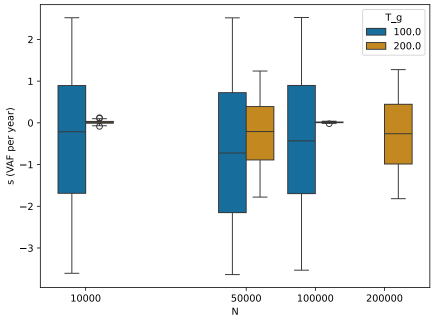

Figure S23: Subject 28

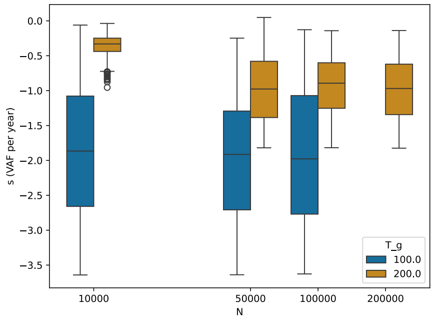

Figure S24: Subject 29

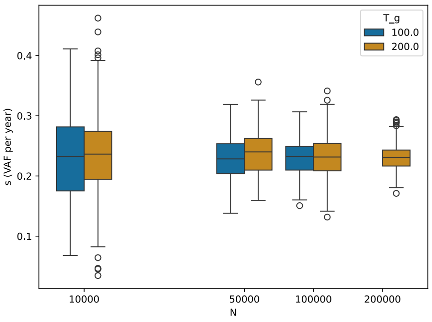

Figure S25: Subject 30

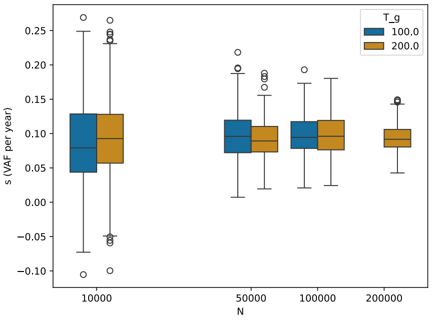

Figure S26: Subject 33

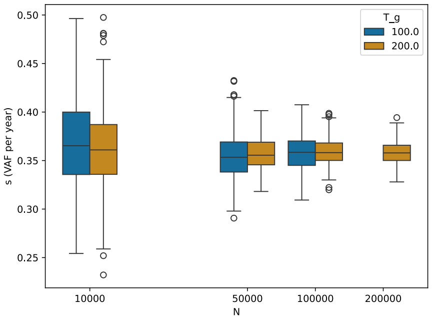

Figure S27: Subject 35

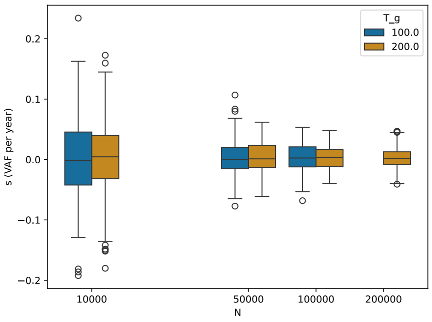

Figure S28: Subject 36

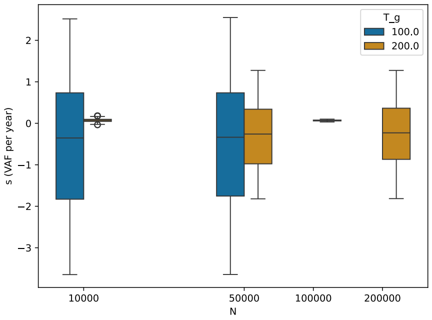

Figure S29: Subject 38

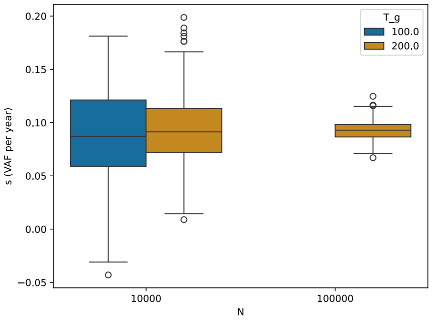

Figure S30: Subject 39

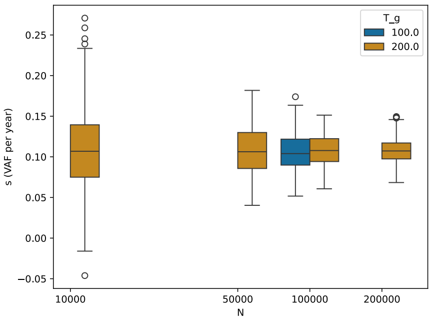

Figure S31: Subject 41

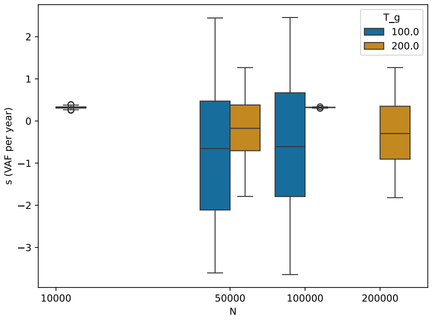

Figure S32: Subject 43

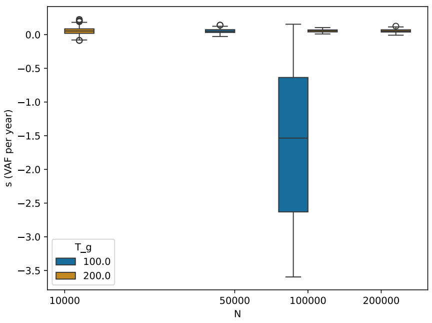

Figure S33: Subject 44

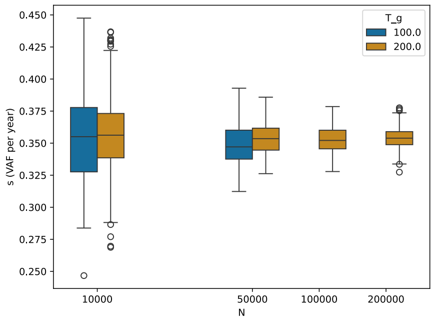

Figure S34: Subject 45

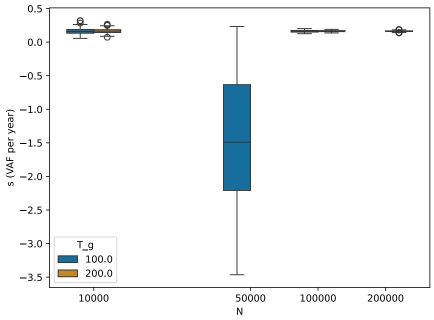

Figure S35: Subject 47

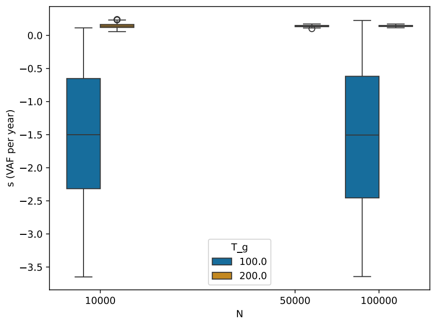

Figure S36: Subject 48

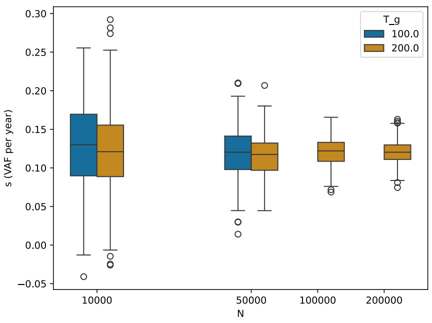

Figure S37: Subject 49

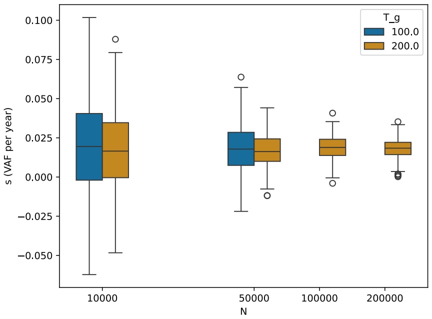

Figure S38: Subject 51

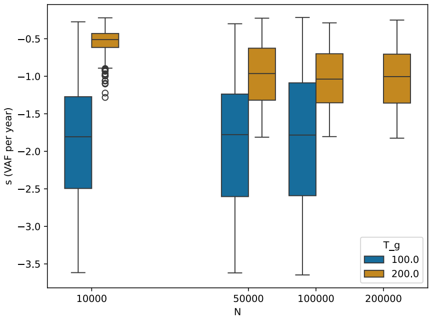

Figure S39: Subject 52

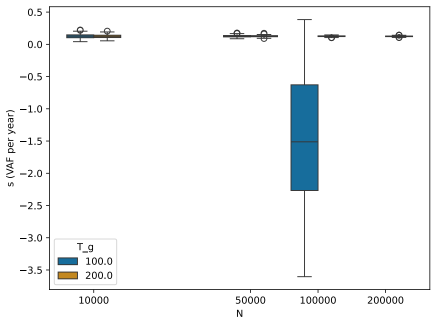

Figure S40: Subject 53

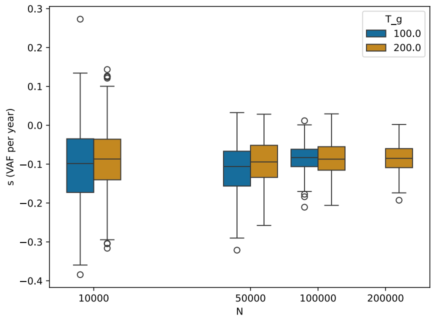

Figure S41: Subject 54

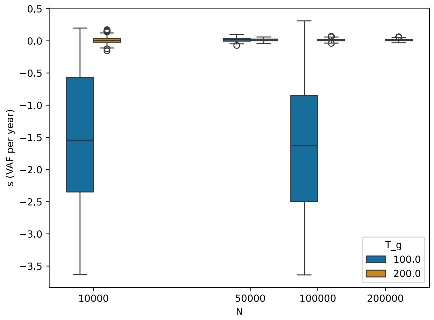

Figure S42: Subject 55

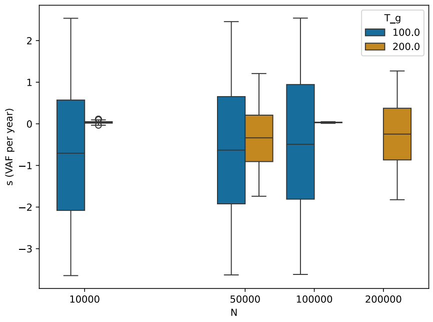

Figure S43: Subject 57

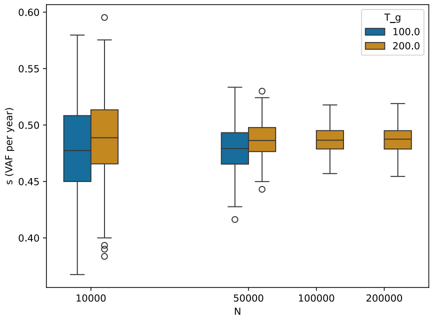

Figure S44: Subject 58

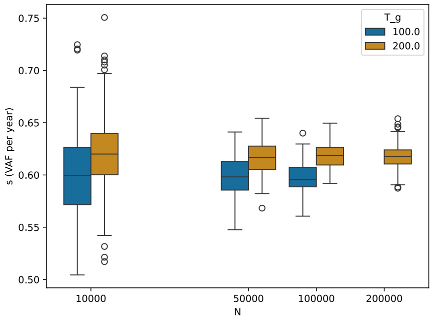

Figure S45: Subject 59

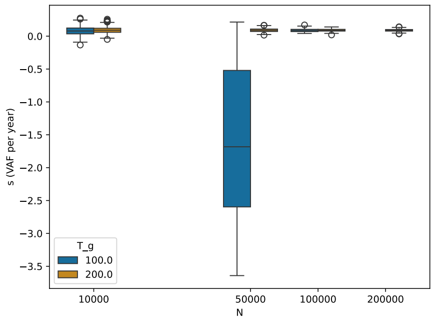

Figure S46: Subject 60

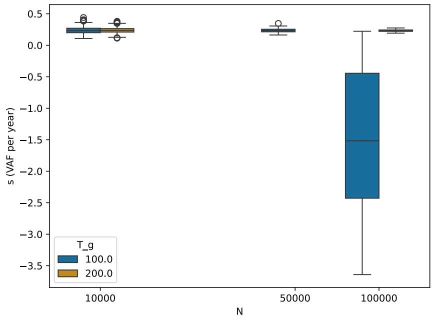

Figure S47: Subject 61

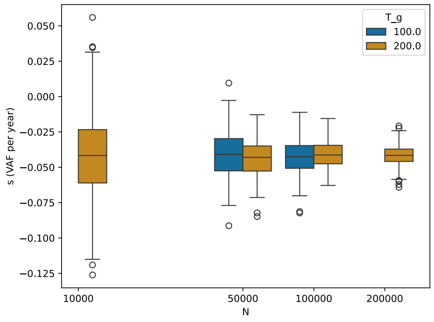

Figure S48: Subject 62

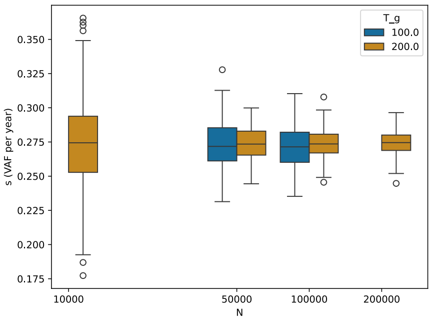

Figure S49: Subject 63

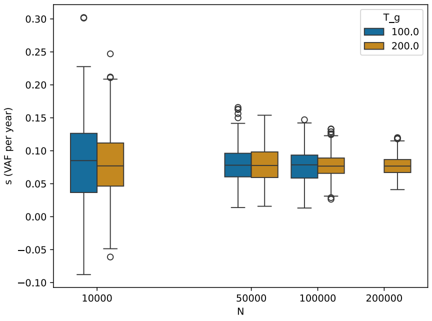

Figure S50: Subject 66

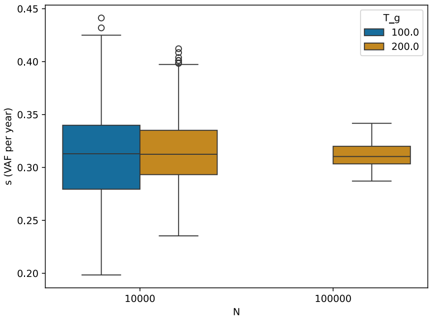

Figure S51: Subject 67

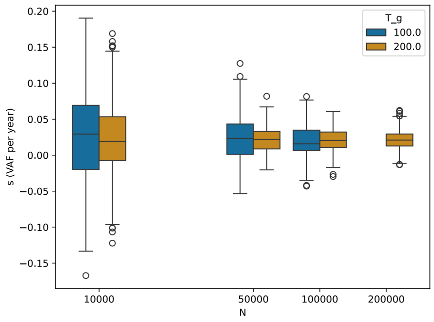

Figure S52: Subject 68

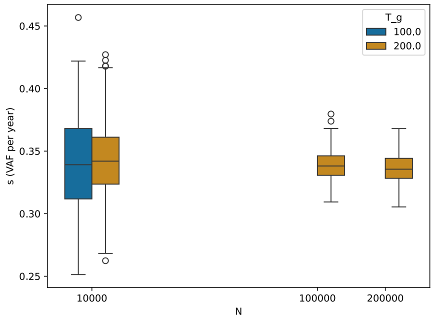

Figure S53: Subject 69

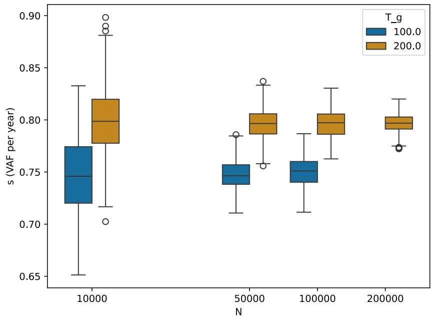

Figure S54: Subject 70

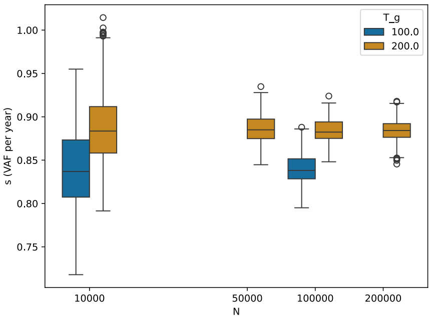

Figure S55: Subject 71

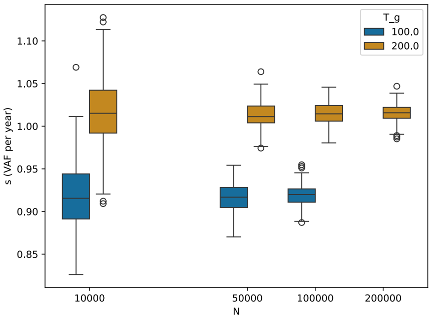

Figure S56: Subject 73

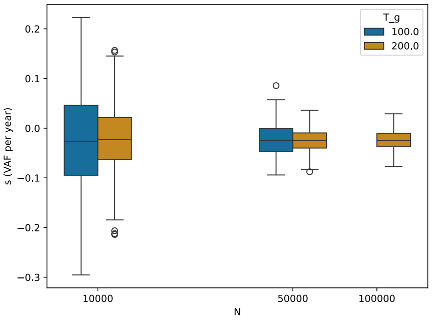

Figure S57: Subject 74

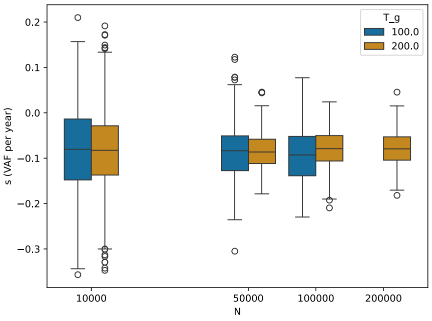

Figure S58: Subject 75

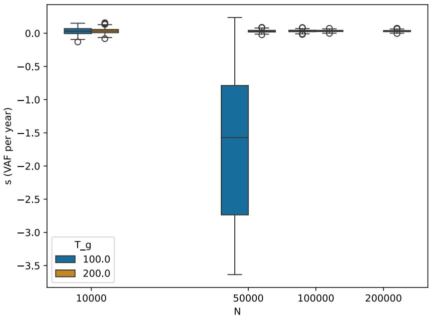

Figure S59: Subject 76

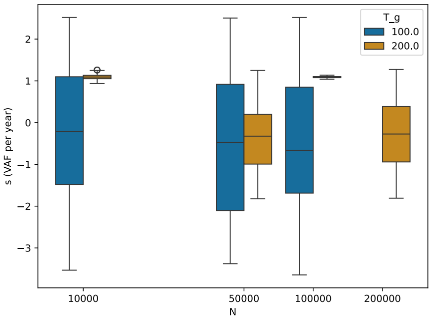

Figure S60: Subject 77

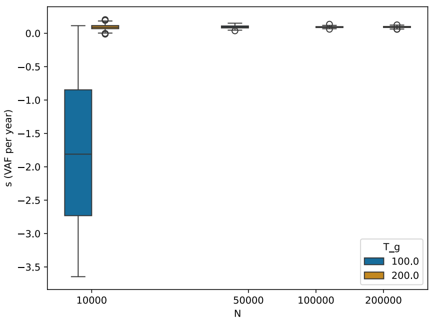

Figure S61: Subject 78

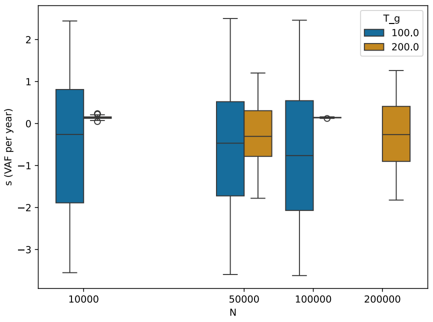

Figure S62: Subject 79

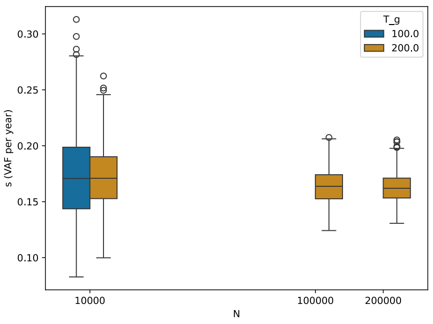

Figure S63: Subject 80

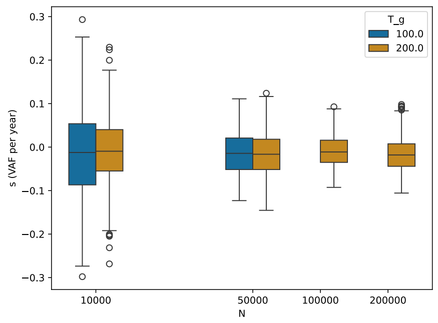

Figure S64: Subject 82

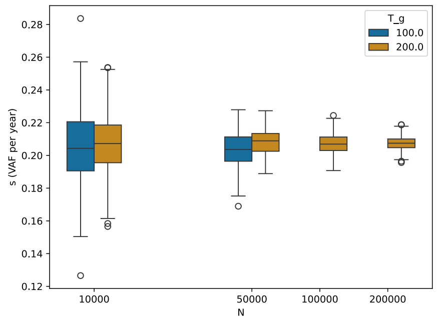

Figure S65: Subject 83

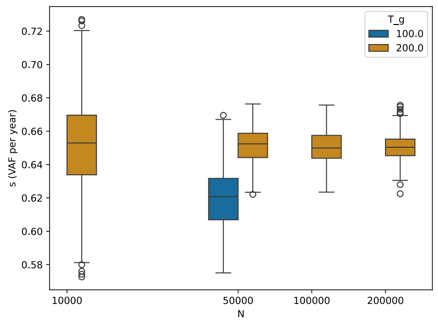

Figure S66: Subject 84

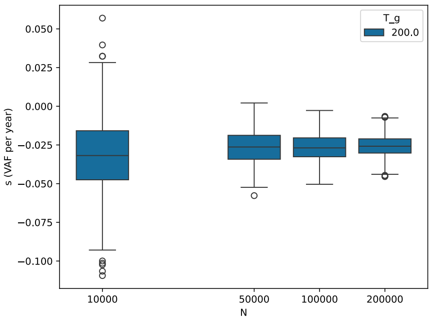

Figure S67: Subject 85

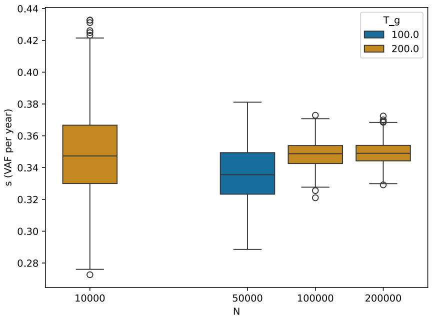

Figure S68: Subject 86

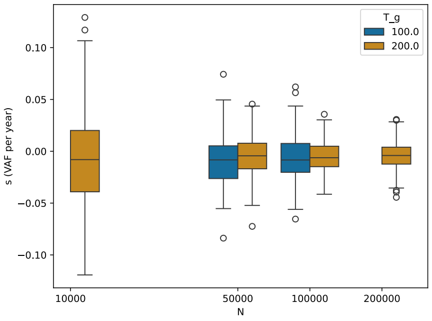

Figure S69: Subject 87

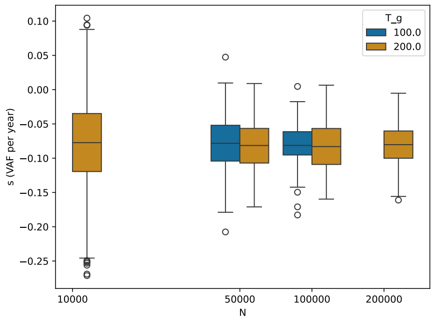

Figure S70: Subject 88

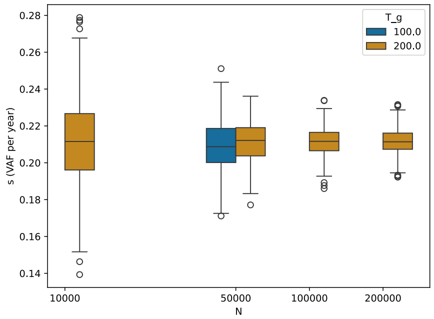

Figure S71: Subject 89

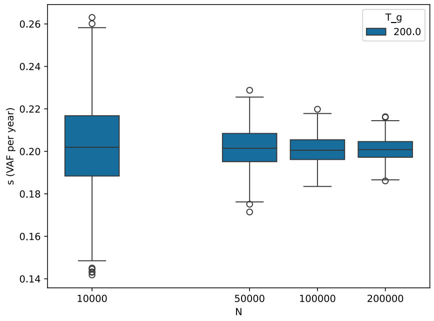

Figure S72: Subject 91

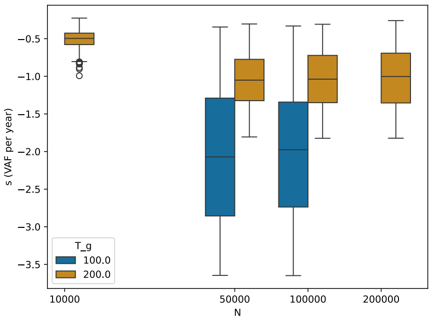

Figure S73: Subject 92

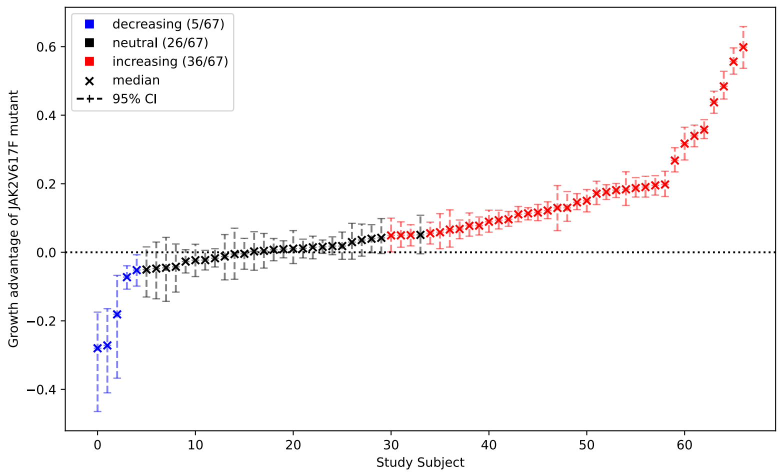

Figure S74: Overview of inferred self-renewal advantage of the *JAK2V617F* mutation in each study subject when assuming $N = 10^4$. Compare to Fig. 3 in the main text.

One clear trend from the collection of figures above is that the spread of the posterior distribution over s is larger for smaller values of N. This means that the statistical significance of the difference of s from zero is less for smaller values of N, i.e. a greater number of subjects would be considered to have a growth rate not significantly different from zero when we assume a smaller value of N. The figure Fig S74 is as Fig. 3 in the main text, but using $N = 10^4$ rather than 10^5.

2.3 Zygocity-related parameters

Our model needs to make some assumptions about the differentiation stages between stem cells and mature cells, since our model operates in terms of stem cells and our data are of mature cells in the peripheral blood. Further, we must make assumptions about the zygocity of mutant cells, as this also impacts the number of mutant alleles we would expect to show up in the peripheral blood.

The key parameters that describe the relationship between the stem cell compartment and the peripheral blood are Δ, k_m, and z, representing the self-renewal advantage of mutation, the (progenitor-to-mature cell) proliferative advantage of mutation, and the fraction of mutated stem cells which are heterozygous. Values of these parameters are difficult to obtain in literature, so we resort to order-of-magnitude estimates when necessary. For Δ we choose 0.017, from Hermange et al. [2]. For k_m, we choose 2 since it stands to reason that the *JAK2V617F* mutation, being associated with overproduction of blood

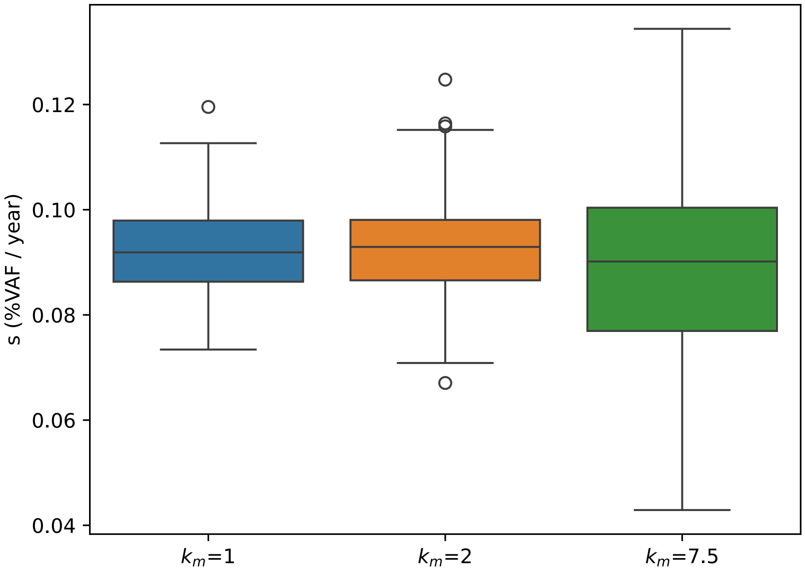

Figure S75: Boxplot depicting posterior distributions of growth rate for three different values of the (progenitor-to-mature) proliferative advantage parameter k_m.

cells, should considerably increase the production of mature blood cells from progenitor cells [1]. There is evidence from mouse models that the impact of the *JAK2V617F* mutation is larger at the progenitor cell level than at the stem cell level [1]. And for z we choose 0.5, since we have very little knowledge of the true zygocity status of most mutant cells and wish to make as neutral an assumption as possible.

Nevertheless, it is natural to wonder about the impact of different choices of these parameter values on our analysis. Rather than run a systematic hyperparameter sweep, we investigate a single case as a simple robstness check. For subject ID 39, we re-ran the inference procedure (assuming $N = 10^5$ and $T_g = 200$ days), for values of $k_m = 1$ and $k_m = 7.5$. The results are depicted in Fig. S75, and we can see that the posterior distribution of the growth rate is centered near the same value for all three cases, with a modest difference in the spread.

We also test, for the same subject, the impact of different choices of the zygocity ratio z. To complement our default assumption of $z = 0.5$, we consider the cases $z = 0$ (all mutant cells are homozygous) and $z = 1$ (all mutant cells are heterozygous). Note that the choice $z = 1$ is only possible when all VAF

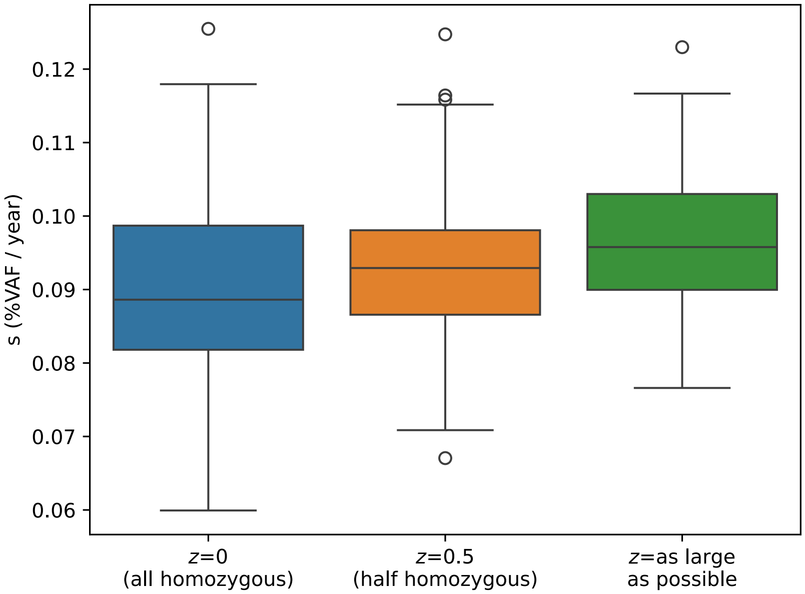

Figure S76: Boxplot depicting the inferred growth rate advantage of *JAK2V617F* clones under different assumptions on the zygocity of mutant cells: all homozygous (left); half homo- and half heterozygous (middle); and all heterozygous (right)

measurements are below 50%, since no more than 50% of the JAK2 alleles would harbor the V617F mutation if all mutant cells were heterozygous. Since all VAF measurements for subject 39 are below 50%, it is possible to use $z = 1$. The results are depicted in Fig. S76. We can see that the inferred growth rate appears slightly higher when more of the cells are inferred to be heterozygous; this is because the larger the fraction of cells that are heterozygous, the greater the clonal fraction must be to achieve the same observed VAF.

3 Mathematical Details

Here we prove the expression for variance of the Moran process as a function of time given a deterministic initial condition, Eq. (5). This proof is known in the community, in particular it can be found on Wikipedia, however the authors are unaware of a published version in the form we are using it. The transition probabilities $P_{i,j} \coloneqq P[X(t+1) = j | X(t) = i]$ for the Moran process under

neutral drift (i.e. $s = 0$) are given by

$$\begin{aligned}
P_{0,0} &= 1 \\
P_{i,i-1} &= \frac{N-i}{N} \cdot \frac{i}{N} \\
P_{i,i} &= 1 - P_{i,i-1} - P_{i,i+1} \\
P_{i,i+1} &= \frac{i}{N} \cdot \frac{N-i}{N} \\
P_{N,N} &= 1
\end{aligned}$$

Notice that $P_{i,i+1} = P_{i,i-1} = i(N-i)/N^2$. Therefore the conditional expectation at the present time step given a deterministic value at the previous time step, $\mathbb{E}[X_t|X_{t-1} = i]$, is

$$\begin{aligned}
\mathbb{E}[X_t|X_{t-1} = i] =&(i-1) \cdot P_{i,i-1} + i \cdot P_{i,i} + (i+1) \cdot P_{i,i+1} \\
=&(i-1) \cdot P_{i,i-1} + i \cdot (1 - P_{i,i-1} - P_{i,i+1}) \\
&+ (i+1) \cdot P_{i,i+1} \\
=&i + P_{i,i+1} - P_{i,i-1} \\
=&i
\end{aligned}$$

Using the law of total expectation, namely that $\mathbb{E}[Y] = \mathbb{E}[\mathbb{E}[Y|Z]]$, we have

$$\begin{aligned}
\mathbb{E}[X_t] &= \mathbb{E}[\mathbb{E}[X_t|X_{t-1}]] \\
&= \mathbb{E}[X_{t-1}]
\end{aligned}$$

which, applied recursively, shows that $\mathbb{E}[X_t] = \mathbb{E}[X_0]$ for all t, and in particular $\mathbb{E}[X_t|X_0 = i] = i$.

To find the variance as a function of time, we will derive a recursion relation expressing the variance at time t in terms of the variance at time $t-1$. Denoting by $V_t = \text{Var}[X_t|X_0 = i]$, we have $V_0 = 0$ and V_1 is

$$\begin{aligned}
V_1 &= \mathbb{E}\left[X_1^2|X_0 = i\right] - \mathbb{E}[X_1|X_0 = 1]^2 \\
&= (i-1)^2 P_{i,i-1} + i^2 P_{i,i} + (i+1)^2 P_{i,i+1} - i^2 \\
&= (-2i+1) P_{i,i-1} + (2i+1) P_{i,i+1} \\
&= P_{i,i-1} + P_{i,i+1} = 2i(N-i)/N^2
\end{aligned}$$

Note that since the transition probabilities don't depend on time, $\text{Var}[X_t|X_{t-1} = i] = V_1$ for all t. Using the law of total variance, which states that $\text{Var}[Y] =$

$\mathbb{E}\left[\mathrm{Var}\left[Y|X\right]\right] + \mathrm{Var}\left[\mathbb{E}\left[Y|X\right]\right]$, we have

$$\begin{aligned}
\mathrm{Var}\left[X_t\right] &= \mathbb{E}\left[\mathrm{Var}\left[X_t|X_{t-1}\right]\right] + \mathrm{Var}\left[\mathbb{E}\left[X_t|X_{t-1}\right]\right] \\
&= \mathbb{E}\left[\frac{2}{N^2}X_{t-1}(N - X_{t-1})\right] + \mathrm{Var}\left[X_{t-1}\right] \\
&= \frac{2}{N^2}\left(N\mathbb{E}\left[X_{t-1}\right] - \mathbb{E}\left[X_{t-1}^2\right]\right) + \mathrm{Var}\left[X_{t-1}\right] \\
&= \frac{2}{N^2}\left(N\mathbb{E}\left[X_{t-1}\right] - \mathrm{Var}\left[X_{t-1}\right] - \mathbb{E}\left[X_{t-1}\right]^2\right) + \mathrm{Var}\left[X_{t-1}\right]
\end{aligned}$$

where we have used $\mathbb{E}\left[X_{t-1}^2\right] = \mathrm{Var}\left[X_{t-1}\right] + \mathbb{E}\left[X_{t-1}\right]^2$. Conditioning on $X_0 = i$ we have

$$\begin{aligned}
V_t &= \frac{2}{N^2}\left(Ni - V_{t-1} - i^2\right) + V_{t-1} \\
V_t &= \frac{2}{N^2}i(N-i) + \left(1 - \frac{2}{N^2}\right)V_{t-1} \\
V_t &= V_1 + \left(1 - \frac{2}{N^2}\right)V_{t-1}
\end{aligned}$$

where we have used $\mathbb{E}\left[X_t|X_0 = i\right] = i$. This is a first-order non-homogeneous recurrence relation for V_t, and it can be solved explicitly as shown below.

Subtracting $N^2V_1/2$ from both sides gives

$$\begin{aligned}
V_t - \frac{N^2}{2}V_1 &= \left(1 - \frac{N^2}{2}\right)V_1 + \left(1 - \frac{2}{N^2}\right)V_{t-1} \\
&= \left(1 - \frac{2}{N^2}\right)\left(V_{t-1} - \frac{N^2}{2}V_1\right)
\end{aligned}$$

Letting $W_t = V_t - \frac{N^2}{2}V_1$, we now have $W_t = (1 - 2/N^2)W_{t-1}$. Since this recurrence relation for W_t is homogeneous, its solution is simply $W_t = (1 - 2/N^2)^{t-1}W_1$. Translating this back into V_t, we get

$$\begin{aligned}
V_t - \frac{N^2}{2}V_1 &= \left(1 - \frac{2}{N^2}\right)^{t-1}\left(V_1 - \frac{N^2}{2}V_1\right) \\
&= -\frac{N^2}{2}V_1\left(1 - \frac{2}{N^2}\right)^t
\end{aligned}$$

and therefore

$$
\begin{aligned}
V_t &= \frac{N^2}{2} V_1 \left(1 - \left(1 - \frac{2}{N^2}\right)^t\right) \\
&= \frac{N^2}{2} \frac{2i(N-i)}{N^2} \left(1 - \left(1 - \frac{2}{N^2}\right)^t\right) \\
&= \tfrac{2i}{N}\left(1 - \tfrac{i}{N}\right) \frac{1 - \left(1 - \frac{2}{N^2}\right)^t}{\frac{2}{N^2}}
\end{aligned}
$$

and we are done.

4 Partial fitting and prediction for all subjects

Of the 67 subjects we consider, thirty four (34) had at least three sequential VAF measurements available. For these subjects, we fit the Moran model to the first two measurements (or the first three if at least four measurements were available), and use the fitted model to predict the subsequent measurements. We require at least three measurements in total because we need at least two training points to obtain a fit with an uncertainty estimate, and at least one testing point to compare against the model prediction. The results are depicted in Figs. S77–S110.

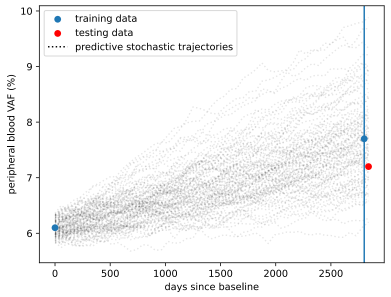

Figure S77: Partial fit and prediction plot for GESUS subject number 10

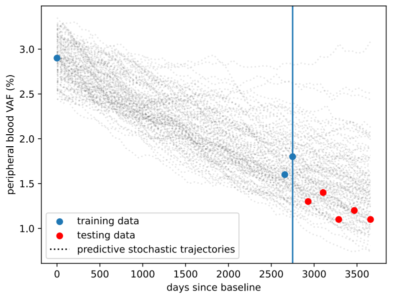

Figure S78: Partial fit and prediction plot for GESUS subject number 12

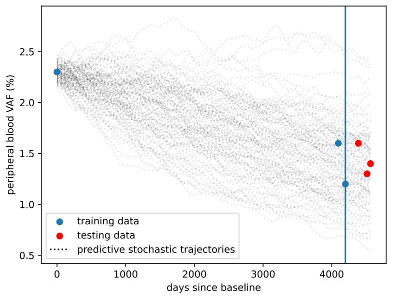

Figure S79: Partial fit and prediction plot for GESUS subject number 13

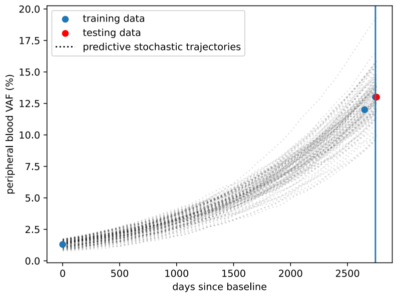

Figure S80: Partial fit and prediction plot for GESUS subject number 15

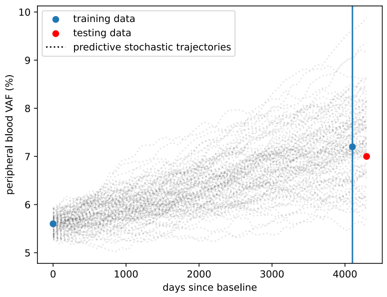

Figure S81: Partial fit and prediction plot for GESUS subject number 17

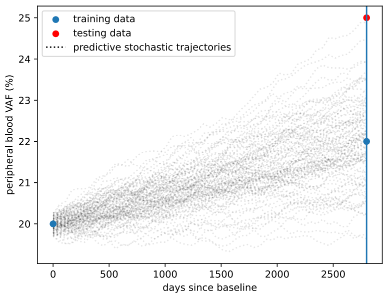

Figure S82: Partial fit and prediction plot for GESUS subject number 1

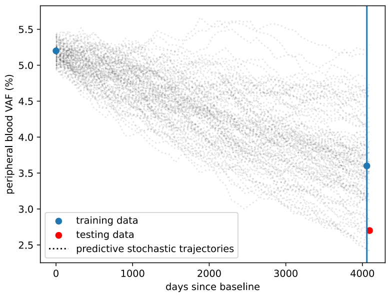

Figure S83: Partial fit and prediction plot for GESUS subject number 21

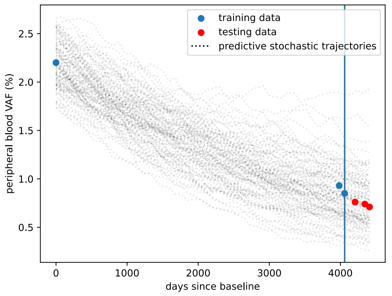

Figure S84: Partial fit and prediction plot for GESUS subject number 25

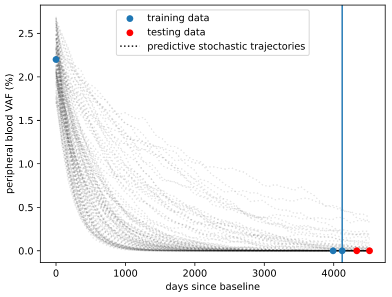

Figure S85: Partial fit and prediction plot for GESUS subject number 29

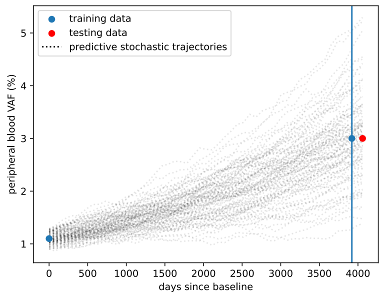

Figure S86: Partial fit and prediction plot for GESUS subject number 33

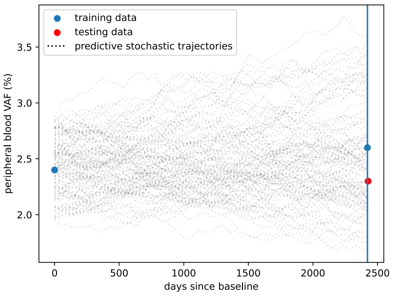

Figure S87: Partial fit and prediction plot for GESUS subject number 36

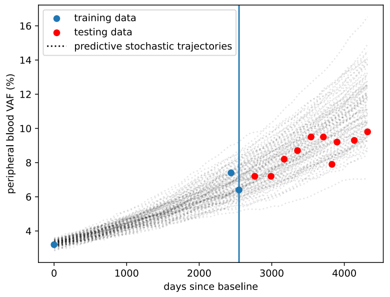

Figure S88: Partial fit and prediction plot for GESUS subject number 39

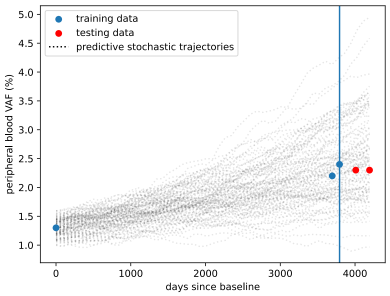

Figure S89: Partial fit and prediction plot for GESUS subject number 44

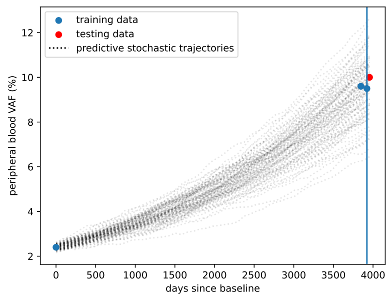

Figure S90: Partial fit and prediction plot for GESUS subject number 48

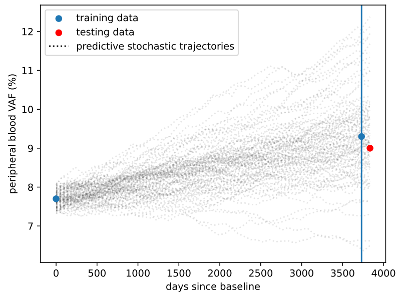

Figure S91: Partial fit and prediction plot for GESUS subject number 51

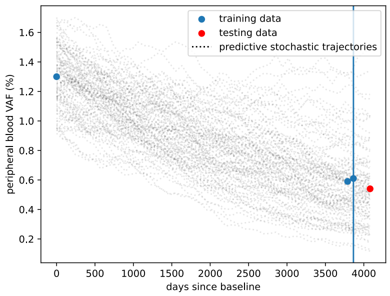

Figure S92: Partial fit and prediction plot for GESUS subject number 54

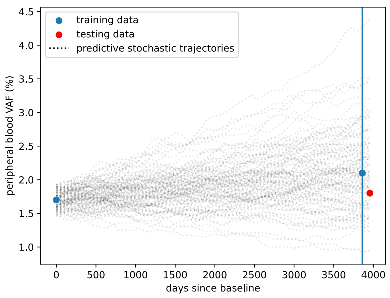

Figure S93: Partial fit and prediction plot for GESUS subject number 55

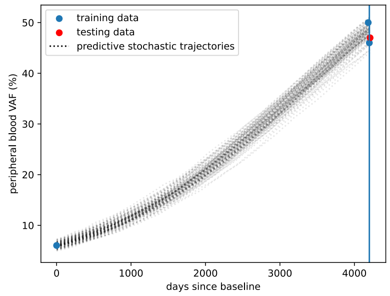

Figure S94: Partial fit and prediction plot for GESUS subject number 5

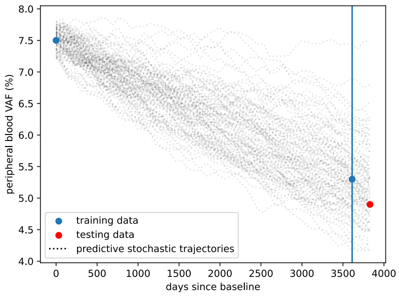

Figure S95: Partial fit and prediction plot for GESUS subject number 62

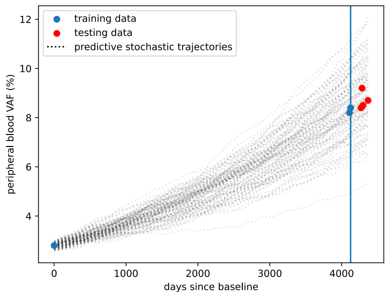

Figure S98: Partial fit and prediction plot for GESUS subject number 6

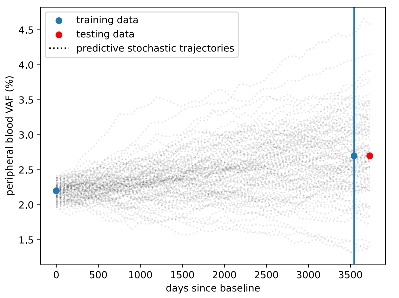

Figure S96: Partial fit and prediction plot for GESUS subject number 68

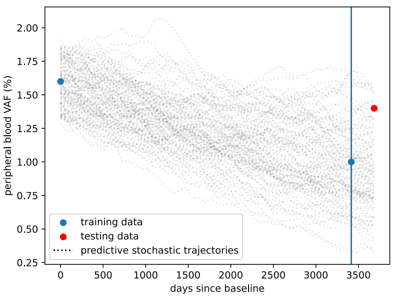

Figure S99: Partial fit and prediction plot for GESUS subject number 74

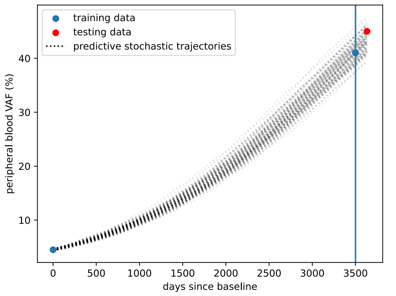

Figure S97: Partial fit and prediction plot for GESUS subject number 69

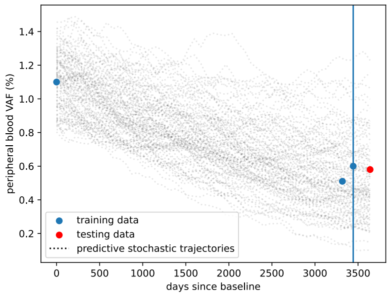

Figure S100: Partial fit and prediction plot for GESUS subject number 75

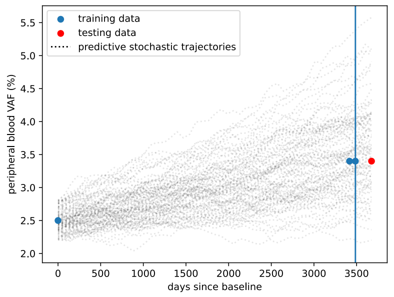

Figure S101: Partial fit and prediction plot for GESUS subject number 76

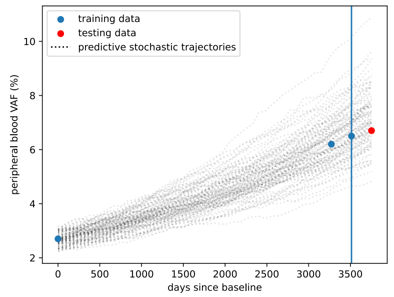

Figure S102: Partial fit and prediction plot for GESUS subject number 78

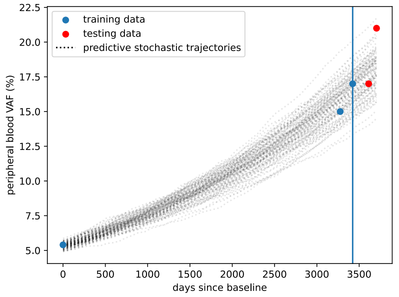

Figure S103: Partial fit and prediction plot for GESUS subject number 80

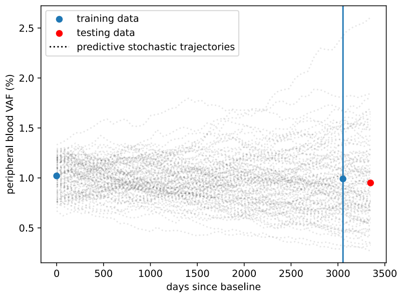

Figure S104: Partial fit and prediction plot for GESUS subject number 82

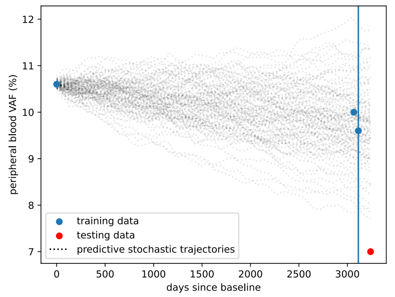

Figure S105: Partial fit and prediction plot for GESUS subject number 85

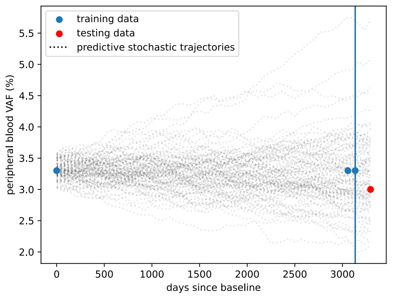

Figure S106: Partial fit and prediction plot for GESUS subject number 87

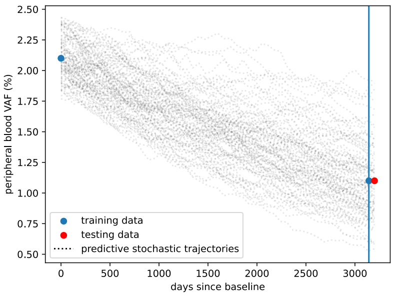

Figure S107: Partial fit and prediction plot for GESUS subject number 88

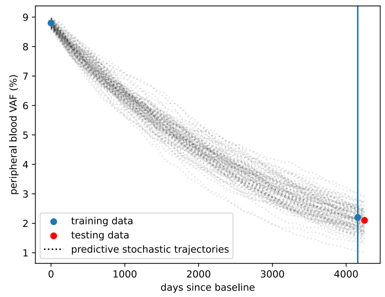

Figure S108: Partial fit and prediction plot for GESUS subject number 8

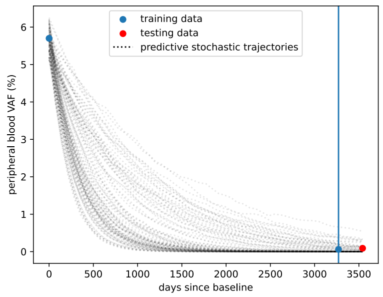

Figure S110: Partial fit and prediction plot for GESUS subject number 92

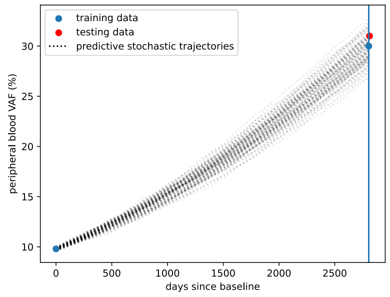

Figure S109: Partial fit and prediction plot for GESUS subject number 91

5 Correlation of growth rate with clinical measurements

We now briefly discuss correlations of the inferred VAF growth rate, s, with other available hematological measurements. Specifically, we consider VAF, monocyte count, thrmobocyte count, and neutrophil-to-lymphocyte ratio (NLR), which has been used as a marker of chronic inflammation and is positively associated with all-cause mortality both with and without MPN disease [4]. The growth rate was computed using all available measurements taken in the absence of treatment, and all other variables were taken at the first followup visit. Paired scatter plots are shown in Fig. S111.

The clearest positive correlation in this figure is between growth rate and VAF. This makes sense, as the VAF measurement used here is from the first followup, and individuals who experience rapid clonal expansion will necessarily have higher VAF at first followup. Other variables show either no or moderate positive correlation with growth rate.

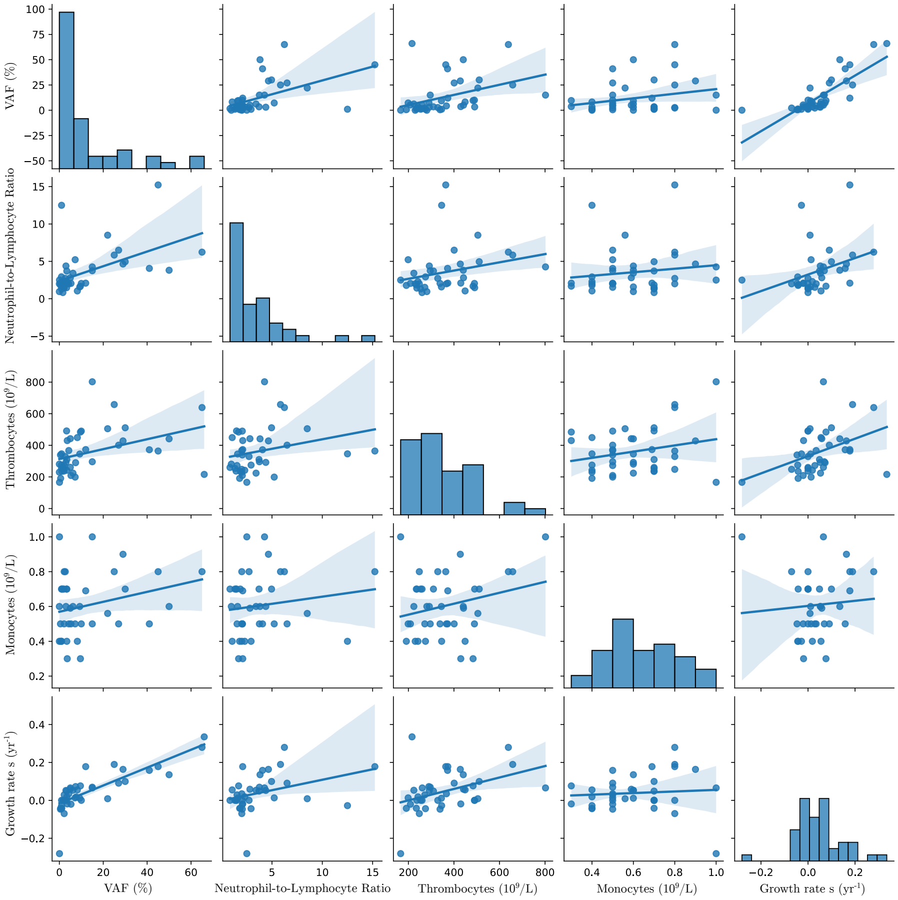

Figure S111: Pair plots depicting correlation between five quantities: VAF (%), Neutrophil-to-lymphocyte ratio (NLR), thrombocyte count ($10^9/L$), monocyte count ($10^9/L$), and inferred growth rate (yr^{-1}). Inferred growth rate is based on all available measurements taken in the absence of treatment for hematological malignancy, and all other variables were taken at the first followup measurement.

References

[1] Shubha Anand, Frances Stedham, Philip Beer, Emma Gudgin, Christina A. Ortmann, Anthony Bench, Wendy Erber, Anthony R. Green, and Brian J. P. Huntly. Effects of the JAK2 mutation on the hematopoietic stem and progenitor compartment in human myeloproliferative neoplasms. *Blood*, 118(1):177–181, July 2011.

[2] Gurvan Hermange, Alicia Rakotonirainy, Mahmoud Bentriou, Amandine Tisserand, Mira El-Khoury, François Girodon, Christophe Marzac, William Vainchenker, Isabelle Plo, and Paul-Henry Cournède. Inferring the initiation and development of myeloproliferative neoplasms. *Proceedings of the National Academy of Sciences*, 119(37):e2120374119, September 2022. Publisher: Proceedings of the National Academy of Sciences.

[3] Marie Hvelplund Kristiansen, Lasse Kjær, Vibe Skov, Morten Kranker Larsen, Christina Ellervik, Hans Carl Hasselbalch, and Troels Wienecke. JAK2V617F mutation is highly prevalent in patients with ischemic stroke: a case-control study. *Blood Advances*, 7(19):5825–5834, September 2023.

[4] Morten Kranker Larsen, Vibe Skov, Lasse Kjær, Christina Schjellerup Eickhardt-Dalbøge, Trine Alma Knudsen, Marie Hvelplund Kristiansen, Anders Lindholm Sørensen, Troels Wienecke, Morten Andersen, Johnny T. Ottesen, Johanne Gudmand-Høyer, Jordan Andrew Snyder, Mikkel Porsborg Andersen, Christian Torp-Pedersen, Henrik Enghusen Poulsen, Thomas Stiehl, Hans Carl Hasselbalch, and Christina Ellervik. Neutrophil-to-lymphocyte ratio and all-cause mortality with and without myeloproliferative neoplasms—a Danish longitudinal study. *Blood Cancer Journal*, 14(1):1–12, February 2024. Publisher: Nature Publishing Group.
